# Nature based solutions for removal of steroid estrogens in wastewater

**DOI:** 10.3389/fmicb.2024.1437795

**Published:** 2024-09-20

**Authors:** Sureka Liyanage, Mark Lay, Graeme Glasgow, Chris Tanner, Rupert Craggs, Grant Northcott

**Affiliations:** ^1^Division of Health, Engineering, Computing and Science, School of Engineering, University of Waikato, Hamilton, New Zealand; ^2^National Institute of Water and Atmospheric Research Ltd, Hamilton, New Zealand; ^3^Consultant, Hamilton, New Zealand

**Keywords:** estrogen, steroid hormones, wastewater treatment, biodegradation, treatment wetland, HRAP

## Abstract

Estrogens are a growing problem in wastewater discharges because they are continuously entering the environment and are biologically active at extremely low concentrations. Their effects on wildlife were first identified several decades before, but the environmental limits and the remedial measures are still not completely elucidated. Most conventional treatment processes were not designed with sufficiently long retention times to effectively remove estrogens. Nature-based wastewater treatment technologies such as treatment wetlands (TW) and high-rate algal ponds (HRAP) are economically feasible alternatives for decentralized wastewater treatment and have promise for removing steroid hormones including estrogens. For small communities with populations below 50,000, the overall cost of TWs and HRAPs is considerably lower than that of advanced decentralized treatment technologies such as activated sludge systems (AS) and sequencing batch reactors (SBR). This results from the simplicity of design, use of less materials in construction, lower energy use, operation and maintenance costs, and operation by non-skilled personnel. The nature-based technologies show high removal (>80%) for both natural and synthetic estrogens. Estrogen removal in TWs can be enhanced using alternative media such as palm mulch, biochar, and construction wastes such as bricks, instead of traditional substrates such as sand and gravel. While TWs are effective in estrogen removal, they have the disadvantage of requiring a relatively large footprint, but this can be reduced by using intensified multilayer wetland filters (IMWF). Using filamentous algae in HRAP (high-rate filamentous algal pond; HRFAP) is an emerging technology for wastewater treatment. The algae supply oxygen via photosynthesis and assimilate nutrients into readily harvestable filamentous algal biomass. Diurnal fluctuations in oxygen supply and pH in these systems provide conditions conducive to the breakdown of estrogens and a wide range of other emerging contaminants. The performance of these nature-based systems varies with seasonal changes in environmental conditions (particularly temperature and solar irradiation), however a greater understanding of operating conditions such as loading rate, hydraulic retention time (HRT), pond/bed depth, dissolved oxygen (DO) concentration and pH, which influence the removal mechanisms (biodegradation, sorption and photodegradation) enable TWs and HRAPs to be successfully used for removing estrogens.

## 1 Introduction

Estrogens are steroid hormones essential for the proper functioning of the endocrine systems of both humans and animals. Steroid estrogens produced within humans or animals, or synthetic analogues administered as contraceptives or hormone replacement therapy, enter sewage treatment plants from daily mammalian excretions. Estrone (E1), 17β-estradiol (E2), and estriol (E3) are natural estrogens produced by all vertebrates, especially females (Ying G. et al., 2002; Manickum and John, 2014). Synthetic estrogens such as ethynylestradiol (EE2) and mestranol (MeEE2) are orally administered as therapeutic drugs for contraceptive birth control. The chemical structures and physiochemical properties of estrogens are given in Table 1. Estrogens are excreted in free forms or as their inactive polar sulphate and glucuronide conjugates, which are later deconjugated into the respective free forms by wastewater-borne microorganisms within wastewater reticulation networks and treatment systems. All humans and animals excrete large quantities of steroid hormones daily within urine and faeces; the average daily excretion of E1, E2, E3, and EE2 per person is 19, 7.7, 81, and 0.41 μg, respectively, (Laurenson et al., 2014). Steroid estrogens are the most prominent estrogenic endocrine disrupting chemical (EDC) in sewage waste, and they detrimentally affect wildlife and possibly humans (Pal et al., 2010). Bisphenol A (BPA; used as a resin in dental products) and nonylphenol (NP; used in household cleaning products; Laganà et al., 2004) are other possible estrogenic compounds present in municipal wastewater. Although they are present at concentrations several thousand times greater than steroid estrogens, their estrogenic potency (the ability to bind to estrogen receptors) is significantly low. The concentrations of common estrogenic EDCs present in sewage water and their estrogenic potency are presented in the Table 2. If estrogens are not completely biodegraded or removed during wastewater treatment, residues of biologically active parent compounds and/or their transformation products can be released into the environment with the discharged effluent.

**Table 1:** Physicochemical properties of estrogens.

Types of estrogens		Name of the estrogen	Molecular structure	Molecular weight (g/mol)^a^	Water solubility (mg/L) at 20°C	Log K_ow_	pK_a_
Free estrogens	Natural estrogens	Estrone (E1)	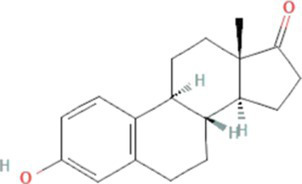	270.4	0.8–12.4^e^	3.1^a^	10.3^b^
		17β-estradiol (E2)	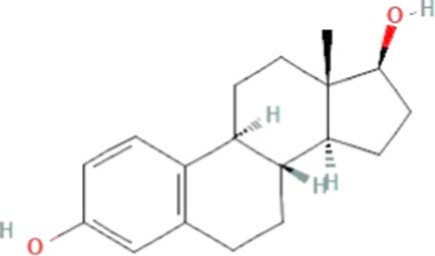	272.4	3.9–13.3^e^	4.0^a^	10.3^b^
		Estriol (E3)	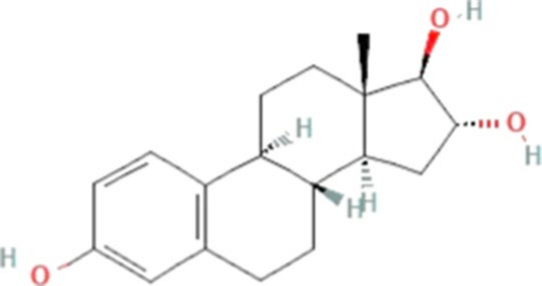	288.4	3.2–13.3^e^	2.4^a^	10.4^d^
	Synthetic estrogens	17α-ethinylestradiol (EE2)	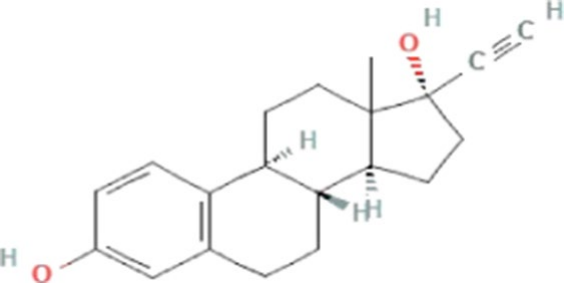	296.4	4.8^f^	3.7^a^	10.3^d^
		Mestranol (MeEE2)	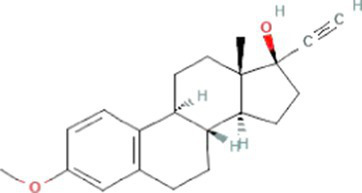	310.4	0.3^f^	4.6^a^	17.6^b^^*^ 13.1^d^
Conjugated estrogens	Sulfate conjugated estrogens	Estrone-3-sulphate (E1-3S)	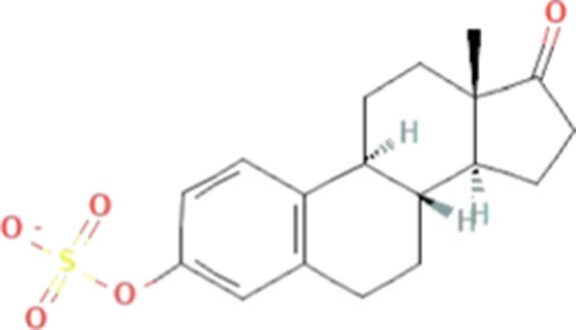	349.4	5.9^b^	0.9^h^	-1.7^b^^*^
		17β-Estradiol-17-sulfate (E2-17S)	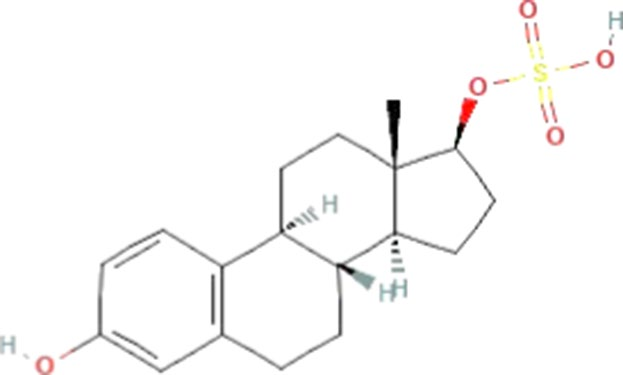	352.4	89,750^g^	1.6^c^	-1.4^g^
	Glucuronide conjugated estrogens	Estrone-3-glucuronide (E3-3G)	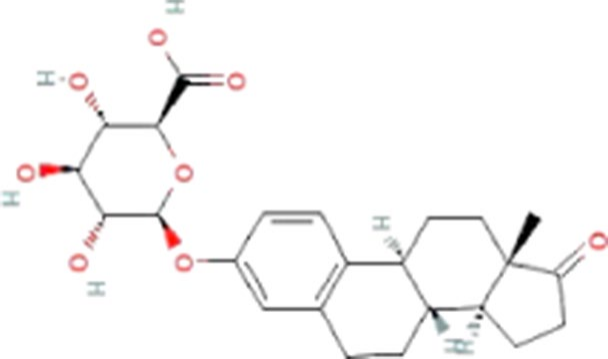	446.5	8,471^g^	1.6^c^	2.8^d^^*^
		Estriol-17-glucuronide (E3-17G)	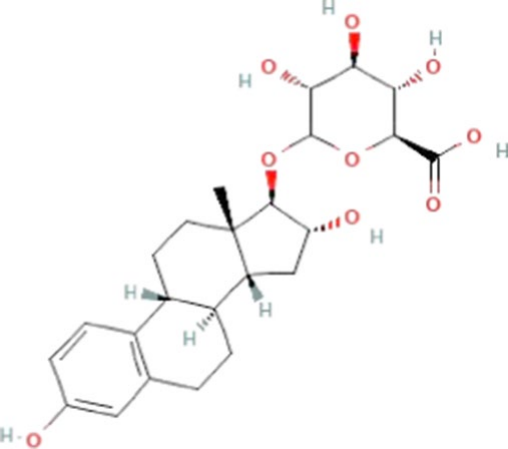	464.5	1.0x10^6g^	1.6^c^	3.5^g^

a Pubchem (2024).

b Drugbank (2024).

c ChemSpider (2024).

d Chemical Book online (2024).

e Hanselman et al. (2003).

f Lai et al. (2000).

g Yu et al. (2019).

h Koh et al. (2008).

* Predicted value.

**Table 2:** Common EDCs present in sewage waste and their estrogenic potency.

EDC	Concentration^a^ (ng/L)	Potency^a^
Estrone (E1)	<LOD–>600	0.01–0.1
Estradiol (E2)	Undetected-199	1.0
Ethinyl estradiol (EE2)	>100	0.8–1.9
Estriol (E3)	0–431	0.01–0.08
Mestranol (MeEE2)	2.7^b^	NDA
Androgens	7.9–635	-
Progesterone	<0.2–904	-
Nonylphenol (NP)	0.7–343 μg/L^c^	1.3E−5^e^
Bisphenol A (BPA)	<0.01b–188^d^ μg/L	2.3E−4^e^

a Vymazal et al. (2015).

b Spengler et al. (2001).

c Ying G.-G. et al. (2002).

d Sánchez-avila et al. (2009).

e Kookana et al. (2003).

The presence of steroidal hormones in treated sewage water has been reported worldwide during last 10–20 years (Zhou et al., 2012; Laurenson et al., 2014). Although the shelf-life of steroid estrogens is several hours to days under laboratory conditions (Hanselman et al., 2003), in surface waters, they are considered as pseudo-persistent as they continuously enter the environment (Kumar et al., 2011). The half-life of estrogens depends on many factors including concentration, type and their physicochemical properties, types of microorganisms and their abundance, physicochemical properties of the matrix and environmental conditions (Adeel et al., 2017). Griffith et al. (2023) reported that the average half-life of E1 and E2 were 74 and 49 h in river water. Jurgens et al. (2002) studied the half-life of estrogens in several United Kingdom rivers and observed half-lives of E1 and E2 ranged between 0.1 to 10.9 and 0.2 to 9 days, respectively, at 20°C when the initial concentration was 100 to 500 μg/L. Synthetic estrogen EE2 persists longer in the environment than natural estrogens. The half-life of EE2 under aerobic conditions is 81 days while the half-life of E2 was two days under the same conditions (Kookana et al., 2003). A half-life of 108 days was also reported for EE2 at a lake site in the USA (Zuo et al., 2013).

Estrogens in the environment, especially in water bodies, are a persistent environmental problem (Bell et al., 2018; Sarma, 2019; Grobelak and Kowalska, 2023) because they act as potent endocrine disrupting compounds (Caldwell et al., 2012; Vymazal et al., 2015). Fish exhibit abnormalities in reproduction when continuously exposed to estrogens (Christiansen, 2002; Solé et al., 2000), including decreased egg and sperm production, reduced gamete quality (Kumar et al., 2011), reduced hatchability (Vieira et al., 2020), and feminization of male fish (Kumar et al., 2011; Vieira et al., 2020) which can lead to the extinction of these fish from surface water bodies (Kidd et al., 2007). Other aquatic organisms such as invertebrates including polyps, snails, and freshwater shrimps (Segner et al., 2003) also exhibit abnormalities related to reproduction when exposed to estrogens. Terrestrial organisms which have close interactions with aquatic systems such as amphibians, frogs, reptiles and turtles are negatively affected when exposed to estrogens (Palmer et al., 1995; Hoffmann and Kloas, 2012). Breast cancers in women, prostate cancers (Adeel et al., 2017) and reduction in sperm count and fertility in males (Wojnarowski et al., 2021) are possible harmful effects in humans.

Environmental regulatory bodies in various countries have implemented initiatives to reduce exposure to and control the environmental impact of estrogens. The European Commission included E1, E2, and EE2 on the watch list in EU-wide water monitoring policies (Commission Implementing Decision (EU) 2018/840, 2018; Council of the European Union-2020, 2020). The United States Environmental Protection Agency (USEPA) also includes E1, E2, EE2 and E3 (Federal Register-United States Environmental Protection Agency, 2016), and recently required public water systems to monitor E1, E2, E3 and EE2 under the Safe Drinking Water Act (SDWA) as a part of the Unregulated Contaminant Monitoring (UCM) program (Drinking Water Contaminant Candidate List 5 – Final, 2022). Maximum limits of EDCs in WWTP effluents are still at the recommendation or proposal level (Commission Implementing Decision (EU) 2018/840, 2018).

In the last few years research has focused on removing emerging contaminants including estrogens from wastewater using different treatment systems, at least to a level below which they are biologically active in aquatic organisms to mitigate associated ecological impacts. Many conventional wastewater technologies such as oxidation ponds, trickling filters (Yang et al., 2009) and septic tanks perform poorly in removing EDCs including estrogens. Servos et al. (2005) observed no removal of E1 and E2 by trickling filters. Installation of centralized sanitation systems is expensive (Shukla et al., 2022) and has high operation and maintenance costs. The available advanced treatment technologies such as advanced oxidation processes (AOPs) including photo-Fenton and ozonation (Klavarioti et al., 2009; Rosal et al., 2010) remove 50 to 80% of steroid hormones from wastewater but require high energy use and have high operation and maintenance costs.

Treatment wetlands (TWs), also known as constructed wetlands, and high-rate algal ponds (HRAPs; Craggs et al., 2003), are two wastewater treatment technologies that are applicable to rural communities in many countries of the world (Kamilya et al., 2023). They were originally designed to remove organic matter, nutrients and pathogenic organisms from wastewater (Norvill et al., 2016), but they have also been found to remove a wide range of organic pollutants including personal care and pharmaceutical products and steroid hormones such as estrogens from human wastewater (Vymazal et al., 2015; Kamilya et al., 2023).

**Figure 1:**
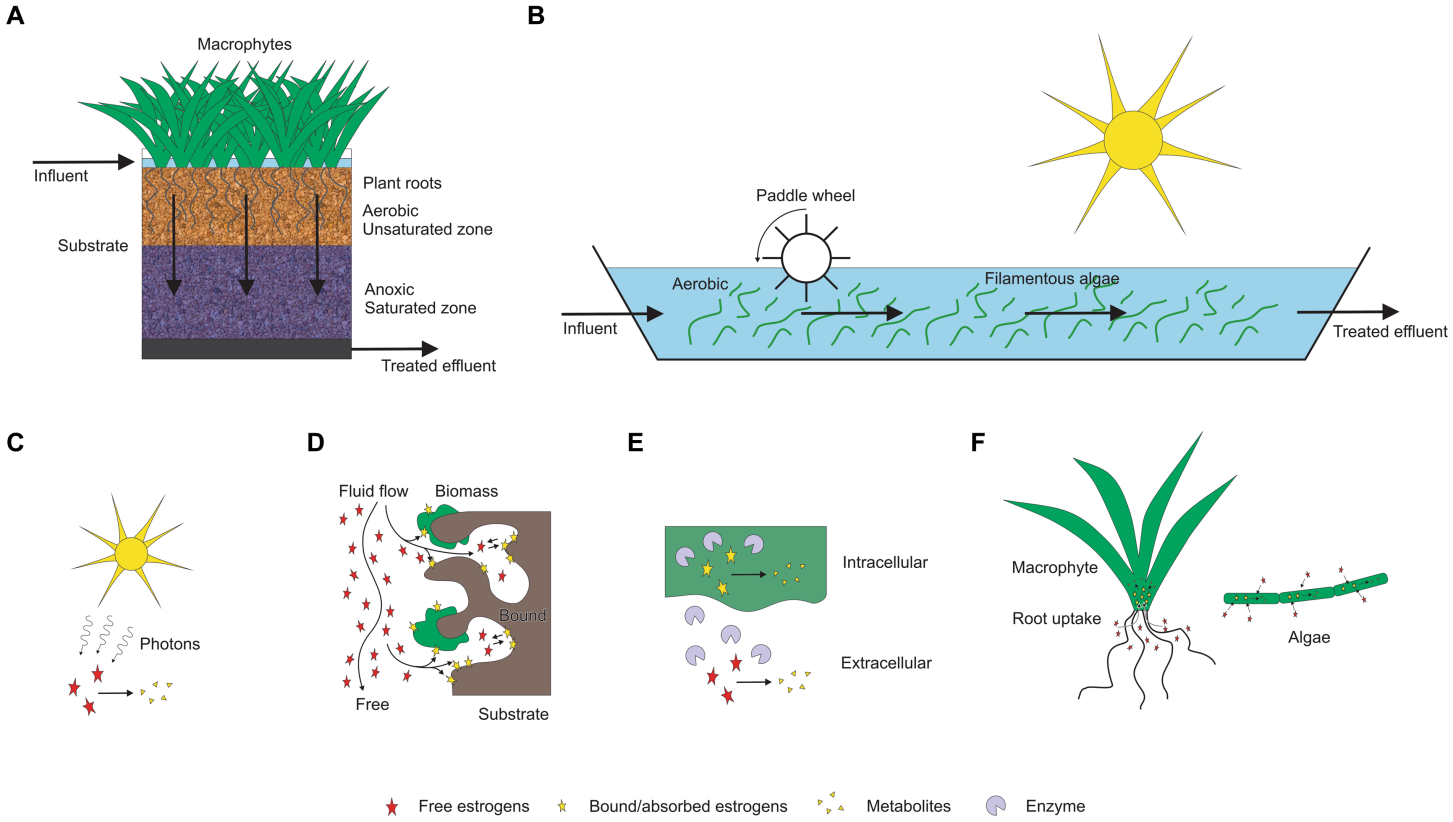
Estrogen removal mechanisms associated in TWs and HRAPs. **(A)** Treatment wetland (TW; **B**) HRAP, **(C)** Photodegradation, **(D)** Adsorption, **(E)** Biodegradation, **(F)** Plant uptake and bioadsorption.

Estrogen removal can occur by multiple mechanisms in TW and HRAP including biodegradation (aerobic and anaerobic), sorption, bioaccumulation, plant-uptake and photodegradation (Figure 1). The reported removal efficacy of steroid hormones by these technologies ranges from 0% (Song et al., 2011) to 100% (Matamoros et al., 2015; Vassalle et al., 2020a; Chen et al., 2021). Variability in performance is attributable to differences in treatment system design and operational conditions including hydraulic loading rate (HLR), hydraulic retention time (HRT), depth of the treatment bed/pond, substrates, microbial community present, pH, ionic strength, estrogen concentration, temperature, dissolved oxygen concentration, light duration and light intensity. This review examines the efficiencies of TWs and HRAPs for estrogen removal, their associated mechanisms, and the influence of operational conditions and environmental factors.

## 2 Treatment wetlands

### 2.1 Types of wetlands and hydrology

There are a wide range of different types of TWs (Fonder and Headley, 2013; Figure 1). In free water surface (FWS) wetlands, which are commonly used after waste stabilisation ponds (Tanner and Sukias, 2003), wastewater flows horizontally through beds of emergent macrophytes rooted in soil. Conditions vary diurnally being mainly aerobic in the surface waters, with anoxic and anaerobic conditions in the plant litter and soils at the base of the wetland (Erler et al., 2011). In subsurface flow (SSF) wetlands, which are most commonly used for on-site and decentralised applications, wastewater passes through the substrate, rather than on top of it. In subsurface horizontal flow (SSHF) wetlands, wastewater flows horizontally through a porous substrate (usually gravel) with emergent macrophytes growing hydroponically in the media. Conditions are predominantly anaerobic in the saturated substrate except near the surface and close to the roots of plants unless tidal flow or artificial aeration is employed (Nivala et al., 2019). In subsurface vertical flow (SSVF) wetlands, the direction of wastewater flow can either be downward [subsurface downward vertical flow (SSDVF)] or upward [subsurface upward vertical flow (SSUVF)]. Most commonly SSDVF utilise sand or fine gravel media which is periodically dosed at the surface with secondary wastewater and then allowed to freely drain. This promotes aerobic conditions suitable for nitrification of ammonium (Stefanakis et al., 2014). Hybrid treatment wetlands (HTW) are formed by connecting different wetland types in series (Vymazal and Kröpfelová, 2015). Intensified multi-layer wetland filters (IMWF) consist of multiple layers of substrate (Nakamura et al., 2017). They may include layers of alternative media (e.g., natural zeolite, shell, and woodchips) and a saturated anoxic zone at the base (Singh et al., 2024). They have a similar treatment capacity to multi-stage vertical flow treatment wetlands (Nakamura et al., 2017) but with a smaller physical footprint (Fonder and Headley, 2013).

**Figure 2:**
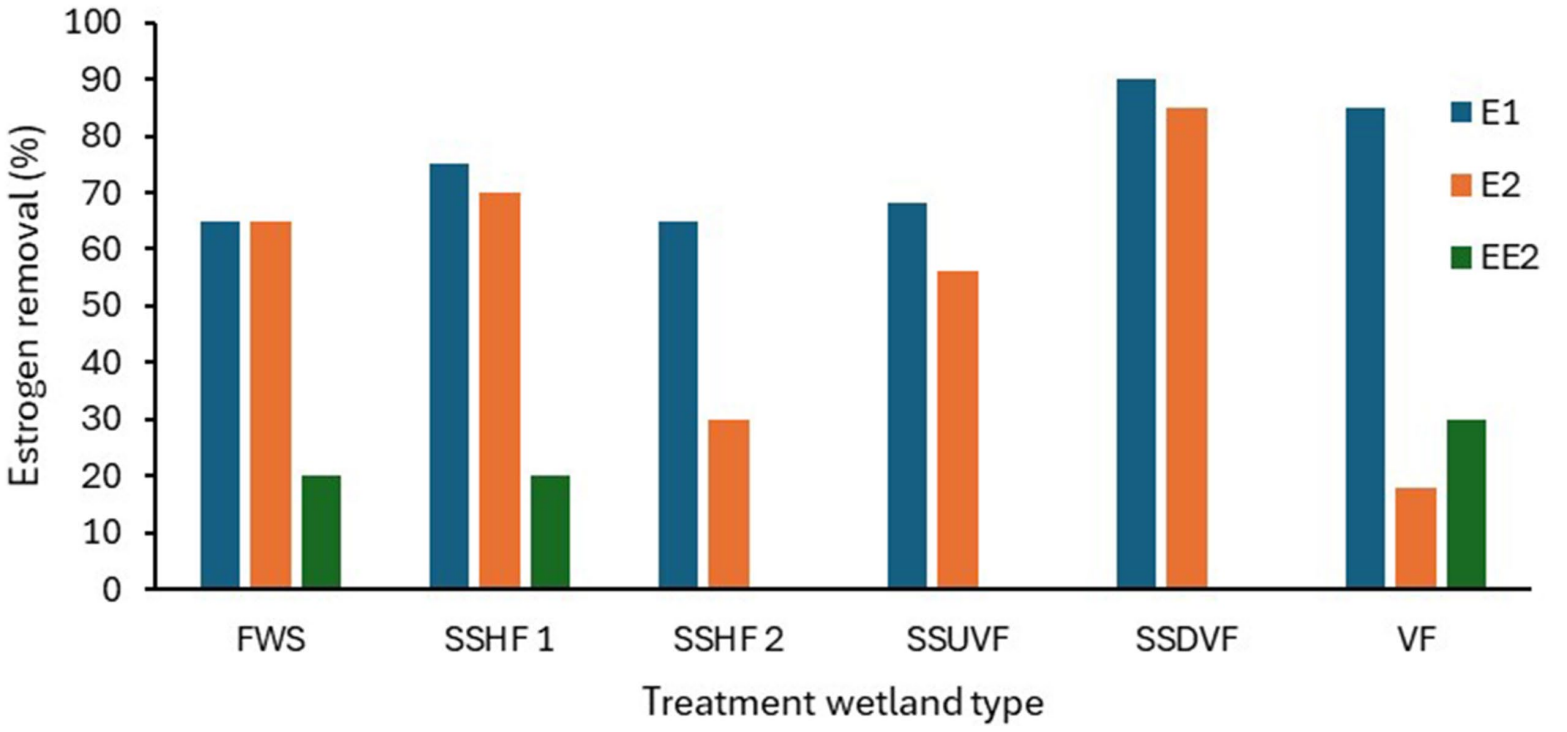
The estrogen removal efficiencies in different TWs based on hydrology. FWS-Free water surface, SSHF-Subsurface horizontal flow, SSUVF-Subsurface upward vertical flow, SSDVF-Subsurface downward vertical flow, VF-Vertical flow (Dai et al., 2016; Herrera-Melián et al., 2018; Ávila et al., 2014).

Most of the estrogen removal studies completed to date on treatment wetlands have mainly focused on single bed TW systems (Ávila et al., 2015; Sharma et al., 2019; David et al., 2022), only few studies were on hybrid TWs, while no studies on the removal of estrogens in wastewater using IMWF has been conducted. The majority have studied estrogen removal with pilot scale TWs using either treated or raw wastewater, the laboratory scale studies have been conducted using synthetic wastewater while a few full-scale studies have been conducted with municipal wastewater. As summarised in Table 3, many studies have been focused on either SSHF or SSVF TWs. SSVF TWS achieved higher estrogen removal efficiencies compared to both SSHF and FWS TWs (Figure 2). For example, Dai et al. (2016) observed higher removal for E1 and E2 by SSHF (75 and 70% respectively) than FWS (65% of both E1 and E2) while the highest removal is by SSDVF TW with 90% of E1 and 81% of E2 removed during summer in 24 h composite samples. Similarly, Herrera-Melián et al. (2018) measured estrogen removal for individual TW units in a hybrid system over 7 days and achieved 85% removal of E1 with VF TW and 63% with SSHF TW. Ávila et al. (2014) studied the efficiency of a hybrid system consisting of two VFTW units, one HFTW unit and one FESTW unit for removing EE2 from treated wastewater and observed a higher removal in VFTWs (30%) than FWS (21%) and SSHF (21%) TWs and a total removal of up to 90%. Although it is difficult to compare results of three studies as operational conditions, type of wastewater and initial concentration of estrogens are different in each study, the estrogen removal efficiencies of the three different types of TWs show a similar pattern. SSDV systems can achieve higher removals of estrogens because the substrate is primarily under aerobic conditions due to intermittent loading increasing biodegradation (Song et al., 2009), while anoxic conditions are prevalent in continuously loaded horizontal flow TWs which reduces biodegradation (Vymazal et al., 2015).

**Table 3:** Estrogen removal efficiencies of different wetlands.

Type of TWs	Type of wastewater	Scale	HLR (L/m^2^/d)	Bed depth (cm)	HRT	Substrate	*Macrophytes*	Initial concentration (ng/L)				Estrogen removal (%)				References
								E1	E2	E3	EE2	E1	E2	E3	EE2	
FWS	Raw domestic sewage	P	~ 1	30	12 h	Vesuvianite, Gravel, Zeolite	*Thalia dealbata, Arundo donax* var. *versicolor*	59 (S)	24 (S)	NA	NA	65 (S), −1 (W)	65 (S), 12 (W)	NA	NA	Dai et al. (2016)
SSHF				60								75 (S), 28(W)	70 (S), 15 (W)			
SSUVF								44 (W)	24 (W)			66 (S), 6 (W)	58 (S), 20 (W)			
SSDVF				60								90 (S), 61 (W)	81 (S), 43 (W)			
SSHF	Municipal wastewater	F	NA	80	7–9 days	Gravel	*Phragmites australis, Phalaris arundinacea*	28–56	6–15	>100	<2–6	>85	>80	>90	NA	Vymazal et al. (2015)
SSHF	Synthetic wastewater	L	NA	NA	2 days	Gravel	*Cyperus isocladus*	NA	NA	NA	16	NA	NA	NA	50	Campos et al. (2019)
						Gravel	*Eichhornia crassipes*								42	
						Gravel	No plant								26	
						Gravel+ bamboo charcoal	*Cyperus isocladus*								96	
					4 days	Gravel	*Cyperus isocladus*	NA	NA	NA	18	NA	NA	NA	43	
						Gravel	*Eichhornia crassipes*								36	
						Gravel	No plant								9	
						Gravel+ bamboo charcoal	*Cyperus isocladus*								81	
2VF	Treated municipal wastewater	P	133	10	4 days	Sand + Gravel	*Phragmites australis*	NA	NA	NA	~5,000	NA	NA	NA	30	Ávila et al. (2014)
SSHF				30	<1 day	Gravel									21	
FWS				10		Gravel									21	
VF	Raw wastewater	P	861	70	~ 3 h	Palm mulch	No plant	ND- 20,000	ND-18,600	424–25,800	ND- 21,700	100	100	100	100	Herrera-Melián (2015)
SSHF			~344	37	3 days	Gravel + sand	*Phragmites australis*									
SSHF			~344	80	~1 h	Volcanic lapilli										
VF			~344	80	~1 h	Lapilli										
VF	Raw domestic wastewater	P	95	70	-	Palm mulch	No plant	95	247	33	NA	85	20	100	NA	(Herrera-Melián et al., 2018)
SSHF (3 units)			142	57 (each)	-	Palm mulch	*Phragmites australis & Cyperus*					63	30	100		
SSHF (2 units)			49	5	-	Ballastic gravel Volcanic lapilli	*Phragmites australis*	~ 25	160	0	NA	100	−42	NA		
SSHF (2 units)						Sand								NA		
VF (2 units)			570	80		Mulch	NA						31	NA	NA	
VF (2 units)			490	80		Gravel							−53	NA	NA	
SSVF	Synthetic sewage (low strength)	L	-	20	12 h	Light expanded clay	*Pistia stratiotes*	NA	NA	NA	200,000	NA	NA	NA	73	Marcelino et al. (2020)
				10		Light expended clay + porcelain tile pieces									49	
				20		Brick pieces									54	
SSHF	Spiked lake water	L	2	12	11 days	polycarbonate (PC)	No plants	NA	NA	NA	5,000	NA	NA	NA	55	Chen et al. (2020)
						PET									45	
						Quartz sand									50	
						Ceramsite (CS)									98	

P, pilot scale; L, laboratory scale; F, full scale; S, summer, W, winter, NA, not analysed.

Hybrid TWs (HTW) can achieve higher removal of estrogens compared to conventional TWs due to the cumulative removal achieved by several wetland units connected in series. Herrera-Melián (2015) obtained complete removal of E1, with a hybrid system consisting of SSHF (three parallel units) followed by a series of three SSHF systems and three VF wetlands.

Although IMWF have not been studied so far for estrogen removal from wastewater, the removal of other organic micropollutants (ibuprofen, naproxen, benzotriazole, diclofenac and carbamazepine) using IMWF has been studied by several researchers (Kahl et al., 2017; Matamoros et al., 2007; Nivala et al., 2018), achieving 77–99% removal compared to 52–98% by municipal WWTP (Sossalla et al., 2021). As estrogens have some similar physicochemical properties to these other micropollutants, for example low solubility in water, IMWF have the potential for enhanced removal of estrogenic steroids from wastewater.

### 2.2 Substrates in treatment wetlands

**Figure 3:**
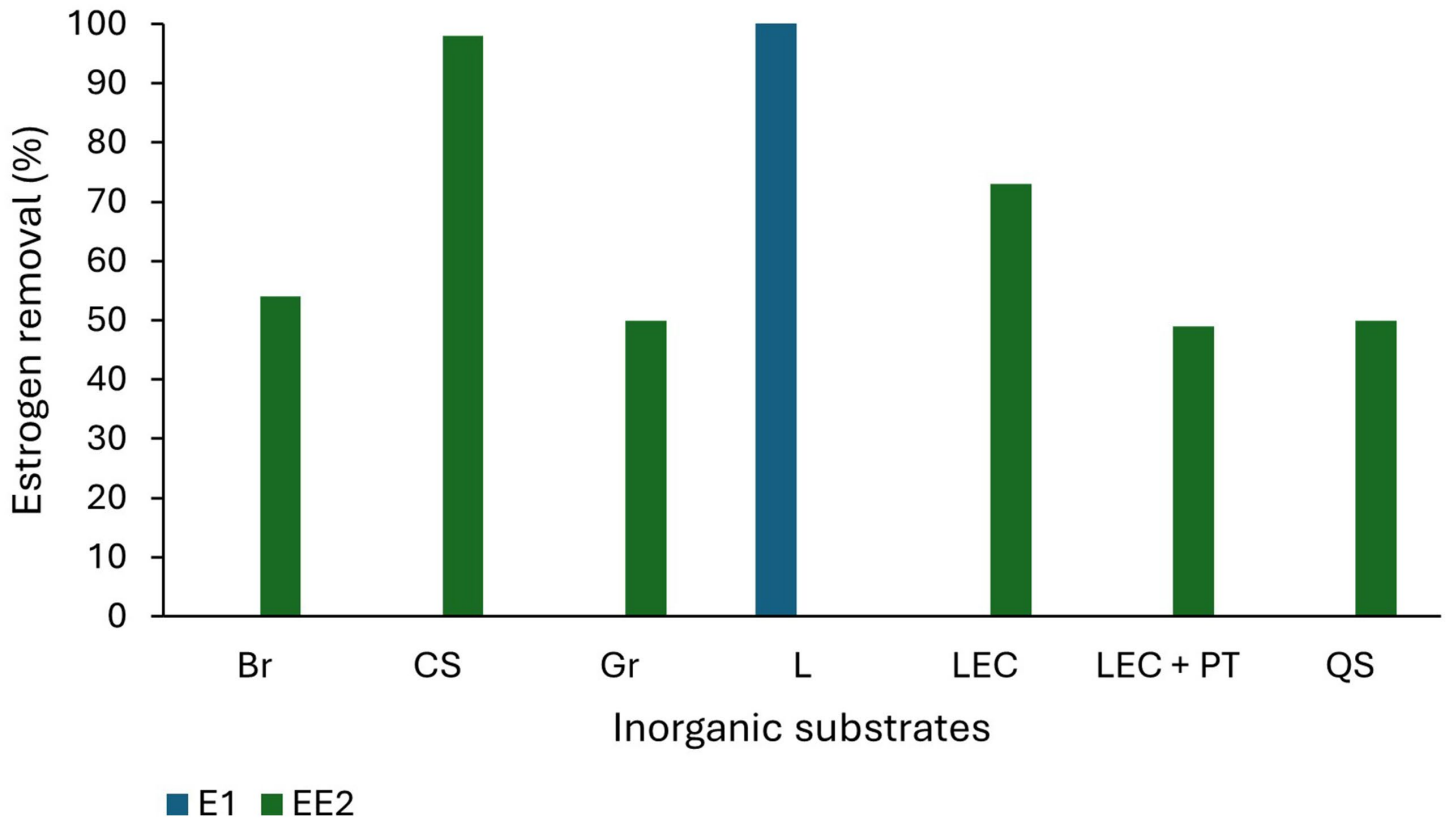
Estrogen removal efficiency of inorganic substrates. Br-Bricks, CS-Ceramsite, Gr-Gravel, L-Lapilli, LEC-Light expanded clay, PT-Porcelain tiles, QS-Quartz sand (Herrera-Melián et al., 2018; Campos et al., 2019; Chen et al., 2020; Marcelino et al., 2020; Kamilya et al., 2023).

Substrates in TWs perform a multifunctional role in estrogen removal (Yang Y. et al., 2018) as they provide substrates for biofilm development and wetland plant growth and act as adsorbents for the sorption of pollutants (Wu et al., 2015). Traditional substrates such as soil, sand, and gravel typically display low estrogen removal capacity (Figure 3) due to their low carbon content and surface area resulting in limited biofilm growth and estrogen adsorption (Song et al., 2009). Substrates with higher organic carbon concentration, for example loam soil (Khanal et al., 2006), mulch and biochar, have higher adsorption capacity and they also support increased biofilm growth enabling greater biodegradation (Kamilya et al., 2023). Substrates with smaller particle size have increased surface area for the adsorption of estrogen (de Mes et al., 2005). Activated carbon displays a very high estrogen removal capability (95–97% for E2 and EE2; Kumar and Mohan, 2011; Ifelebuegu et al., 2015) due to its high porosity and surface area (Guerrero-Gualan et al., 2023) and has been the most common adsorbent (Almazrouei et al., 2023) used in steroid hormone removal from water and wastewater (Ifelebuegu et al., 2015). Dai et al. (2016) studied E1 removal using gravel, vesuvianite (sorosilicate mineral) and zeolite as substrates and observed gravel and vesuvianite had similar removal (0 to 75%) and performed better than zeolite (0 to 35% removal) which removes cations but is less suitable for the removal of neutral and anionic compounds in wastewater.

**Figure 4:**
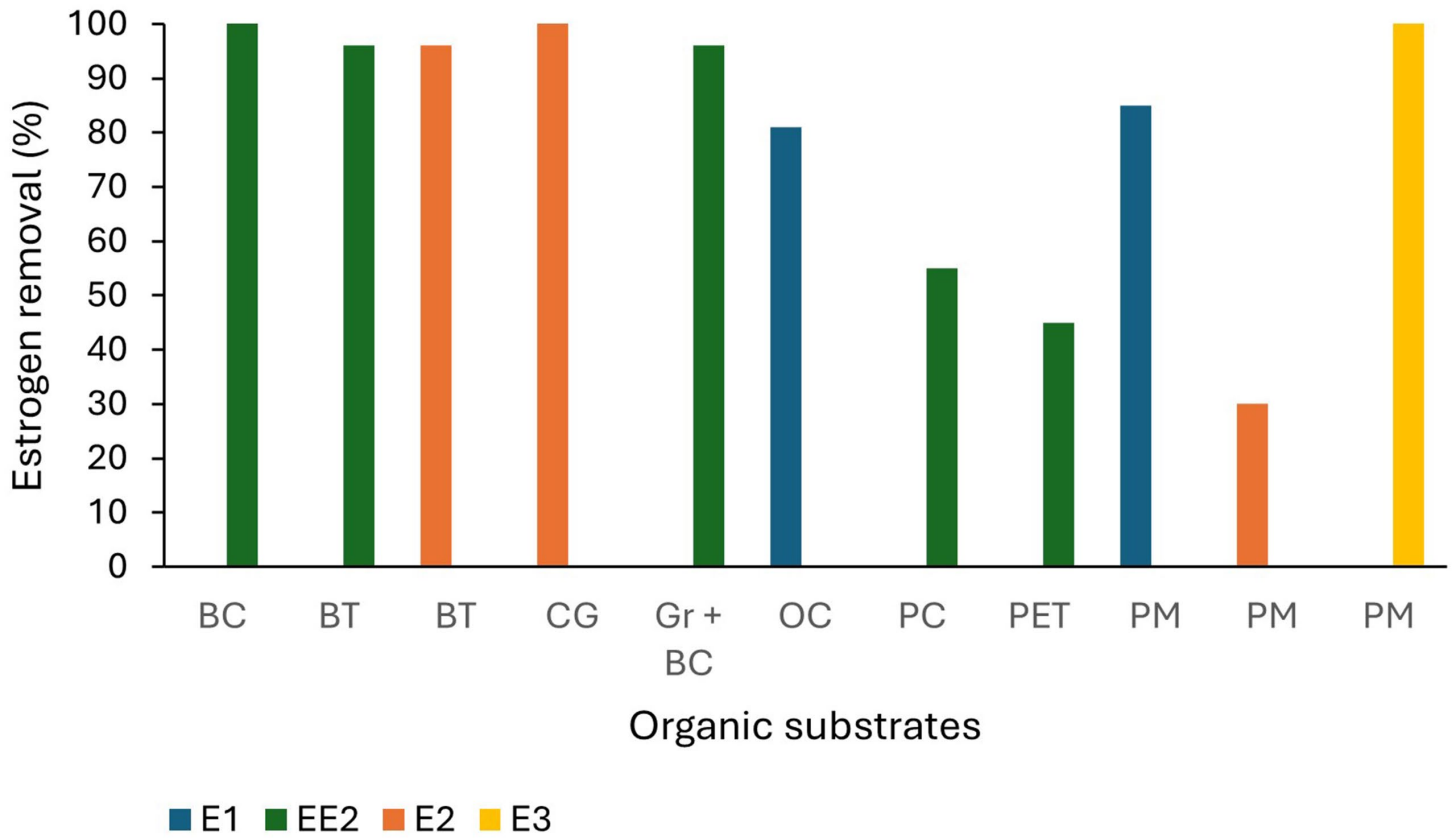
Estrogen removal efficiency of organic substrate materials. BC-Bamboo charcoal, BT-Waste black tea, CG-Coffee ground, OC-Organo clay, PC-Polycarbonate, PET-Polyethylene terephthalate, PM-Palm mulch (Cai et al., 2013; Loffredo et al., 2016; Ifelebuegu et al., 2015; Herrera-Melián et al., 2018; Campos et al., 2019; Marcelino et al., 2020; Chen et al., 2020).

Many alternative substrates have been recently studied for use in TW and most of them have shown high estrogen removal from wastewater (Figure 4). Organoclay (81%; Cai et al., 2013), lapilli (volcanic pyroclastic debris; 100% removal of E1; Kamilya et al., 2023), biochar, spent coffee grounds (100% removal of E2; Loffredo et al., 2016), waste black tea (96% removal of E2 and EE2; Ifelebuegu et al., 2015), palm mulch (complete removal of E3, 85 and 30% removal of E1 and E2; Herrera-Melián et al., 2018), bamboo charcoal mixed with gravel (88% removal of EE2; Campos et al., 2019), construction waste including, bricks, expanded clay aggregates and expanded clay with porcelain tiles (73, 64 and 76% removal of EE2 from wastewater respectively; Marcelino et al., 2020), and plastics [polycarbonate, polyethylene terephthalate (PET)], quartz sand and ceramsite (EE2 was partially removed from spiked lake water; Chen et al., 2020). The removal efficiencies of estrogens by different substrates are summarised in Table 3.

### 2.3 Macrophytes in treatment wetlands

Macrophytes are an essential component in TWs as they act as substrates for microorganisms to grow on, an oxygen provider for soil substrate, a filter for pollutants and they also uptake organic micropollutants (Valipour and Ahn, 2016) with partition coefficients (log K_ow_) between 0.5 and 3 from contaminated waters (Dordio and Carvalho, 2013; Kamilya et al., 2023) directly from wastewater. As free estrogens have log K_ow_ values between 2.4 to 4.6 and the log K_ow_ of conjugated estrogens are between 0.5 and 3, uptake of many estrogens by plants are possible. As the microbial community in the rhizosphere (microenvironment around plant roots) is highly variable depending on the type of plant and the root system, the removal efficiency of pollutants including estrogens also depends on the type of plant (Polinska et al., 2021) and plant density. Including plants in TW can help boost estrogen removal, in some cases by absorbing up to 40% of the total estrogens (Gray and Sedlak, 2005; Hakk et al., 2018).

A generalized guideline for selection of the most suitable plants for estrogen removal does not exist, but obviously one would naturally first consider plants with high removal efficiencies, followed by their ability to tolerate the environmental conditions in which the TWs operate. In general, plants in TWs should be able to tolerate the water-logged/anoxic conditions and high eutrophic conditions, be readily adaptable to changing environments and extreme conditions (Kaur et al., 2020). Different species of *Phragmites* (reed), *Typha* (cattail) and *Scripus* (bulrushes) have been extensively used in TWs around the world (Vymazal, 2011) mainly because they have a high growth rate, can adapt to variable water depth, tolerate cold weather and perform well throughout the year (Valipour and Ahn, 2016). Although the removal efficiency does not depend directly on one factor, TWs planted with *Phragmites* sp. have shown higher estrogen removal efficiency (Kamilya et al., 2023). *Phragmites australis* has been used in most of the research on removal of estrogens by macrophytes and has removed up to 51–81% of E1, E2 and EE2 (Gray and Sedlak, 2005; Song et al., 2011; Vymazal et al., 2015). Herrera-Melián (2015) achieved 100% removal of E1 from raw domestic wastewater in pilot scale SSHF and VF TWs. The estrogen removal capacity of *Phragmites* sp. can be attributed to an extensive root system which supports substantial microbial growth (Valipour and Ahn, 2016) resulting in high removal of trace organic pollutants from wastewater (Liu and Carr, 2013). Plants other than *Phragmites* sp. also have shown high estrogen removal in TWs. For example, *Cyperus isocladus* and *Eichhornia crassipes* removed 96% of EE2 from synthetic wastewater under laboratory scale (Campos et al., 2019).

## 3 High-rate algal ponds (HRAP)

High-rate algal ponds (HRAP) are shallow, paddlewheel mixed, raceway ponds that were primarily developed to remove BOD and nutrients in wastewater by growing algal biomass (Park et al., 2011; Craggs et al., 2014; Norvill et al., 2016). Pollutant removal in HRAPs is mainly by assimilation into algal biomass (Young et al., 2017). HRAP naturally select for algal strains that are able to tolerate the widely varying environmental conditions each day including sunlight, temperature, pH and DO (Weissman et al., 1988). A consortium of species grows in the HRAP, often with a predominant species, which changes periodically with season, operational changes, or under the influence of grazing by zooplankton (Sutherland et al. 2014; Matamoros et al., 2015). Algae grow symbiotically with bacteria (Matamoros et al., 2015, Agüera et al., 2020) and produce oxygen by photosynthesis that promotes the degradation of organic matter by heterotrophic bacteria to CO_2_ providing nutrients and CO_2_ for algal growth (Agüera et al., 2020; Oruganti et al., 2022). The algae that predominate in HRAP can switch their metabolism from autotrophy to either heterotrophy or mixotrophy (Silva et al., 2019) depending on the prevailing environmental conditions. Micropollutants including pharmaceutical organic contaminants, personal care products, EDCs such as steroid hormones, surfactants, pesticides, flame retardants and industrial additives have all been removed by consortia of algae and bacteria (Zhang et al., 2014; Hom-Diaz et al., 2015; Matamoros et al., 2015; Xiong et al., 2018; Li et al., 2019).

HRAPs are an attractive option for wastewater estrogen removal because several studies have shown effective removal ranging between 20 to 100% across a broad estrogen concentration range from 82 ng/L to 16,000 μg/L (Table 4). Estrogen removal efficiencies by algae have been studied on different wastewaters including domestic wastewater (Wang et al., 2020), synthetic wastewater (Ruksrithong and Phattarapattamawong, 2019) and treated wastewater (Solé and Matamoros, 2016; Bai and Acharya, 2019) with most of the studies limited to laboratory scale. *Chlorella* and *Scenedesmus* have been the most common microalgae used in estrogen removal studies and have shown high removal efficiencies under laboratory scale. Pazmino-sosa et al. (2024) observed 80% removal of E1 and E2 by *Scenedesmus* and *Chlorella* cultures spiked with 100 μg/L E1 and E2 (Table 4). *Desmodesmus* have shown 85–90% removal of E2 from domestic wastewater at 1,000 μg/L in 3 days (Wang et al., 2020).

**Table 4:** Estrogen removal efficiency of freshwater algae.

Sample type	Treatment volume (mL)	Scale of study	Name of algae	Algae concentration	Light: dark period (h)	Removal time	Removal mechanism	Initial concentration (μg/L)				Removal efficiency (%)				Reference
								E1	E2	EE2	E3	E1	E2	EE2	E3	
Domestic wastewater	150	L	*Desmodesmus*	6.25 × 10^6^ cells/ml	12:12	3 days	Total removal	NA	1,000	NA	NA	NA	85–99	NA	NA	Wang et al. (2020)
							Photodegradation	NA		NA	NA	NA	<20	NA	NA	
Secondary-treated wastewater	2,000	L	*Chlorella Nitzschia acicularis*	80 mg/L (dry weight)	12:12	10 days	Total removal	NA	NA	10	NA	NA	NA	97	NA	Solé and Matamoros (2016)
							Bioadsorption	NA	NA			NA	NA	<5	NA	
Sterilized treated wastewater	750	L	*Nannochloris*	2.0 × 10^5^ cells/ml	Light (24 h)	7 days	Algae mediated degradation	34–192			NA	29	60		NA	Bai and Acharya (2019)
Primarily treated raw sewage water	20,300	P	Micro algae	Not given	Not given	7 days	Total removal	148 ng/L	82 ng/L	49 ng/L	54 ng/L	55	7	117	42	Vassalle et al. (2020b)
Spiked algal cultures	100	L	*Scenedesmus obliquus*	3×10^6^ cells/ml	12:12	12 days	Total removal	NA	NA	100	NA	NA	NA	80	NA	(Pazmino-sosa et al., 2024)
										300				90		
			*Chlorella vulgaris*				Total removal			100				80		
										300				33		
Spiked algal cultures	350	L	*Chlorella* sp.*, Merismopedia, Closteriopsis, Scenedesmus*	30% of inoculum	Not given	12 days	Degradation	NA	16,000	NA	NA	NA	92	NA	NA	Bano et al. (2021)
Synthetic piggery wastewater	1,000	L	*Scenedesmus obliquus*	100 mg/L (dry weight)	Light	6 h	Adsorption	1 to 5		NA	NA	11	9	NA	NA	Ruksrithong and Phattarapattamawong (2019)
			*Chlorella vulgaris*									10	14			
	5,000		*Scenedesmus obliquus*		Light	5 days	degradation	5				91	99			
			*Chlorella vulgaris*									52	99			
Wastewater	1,000	L	*Chlorella vulgaris*	70 mg/L	16:08	11 days	Adsorption	2		NA	NA	−6	0	NA	NA	Prosenc et al. (2021)
			*Algal bacterial mix culture*			11 days						100	97–98			
			*Chlorella vulgaris*			1 h	Biodegradation					66	99			
			*Algal bacterial mix culture*			4 days						100	100			
Wastewater effluent	135,000	P	*Spirogyra*	~1.90 g /L (dry weight)	16:08	Not given	Total removal	4	3	2	NA	42	24	55	NA	Song et al. (2011)
Treated wastewater	2,500	L	*Spirogyra*	4 mg/L (fresh weight)	12:12	20 days	Total removal	NA	NA	100	NA	NA	NA	94	NA	Garcia-Rodríguez et al. (2015)

P, pilot scale; L, laboratory scale; F, full scale; NA, not analysed.

**Figure 5:**
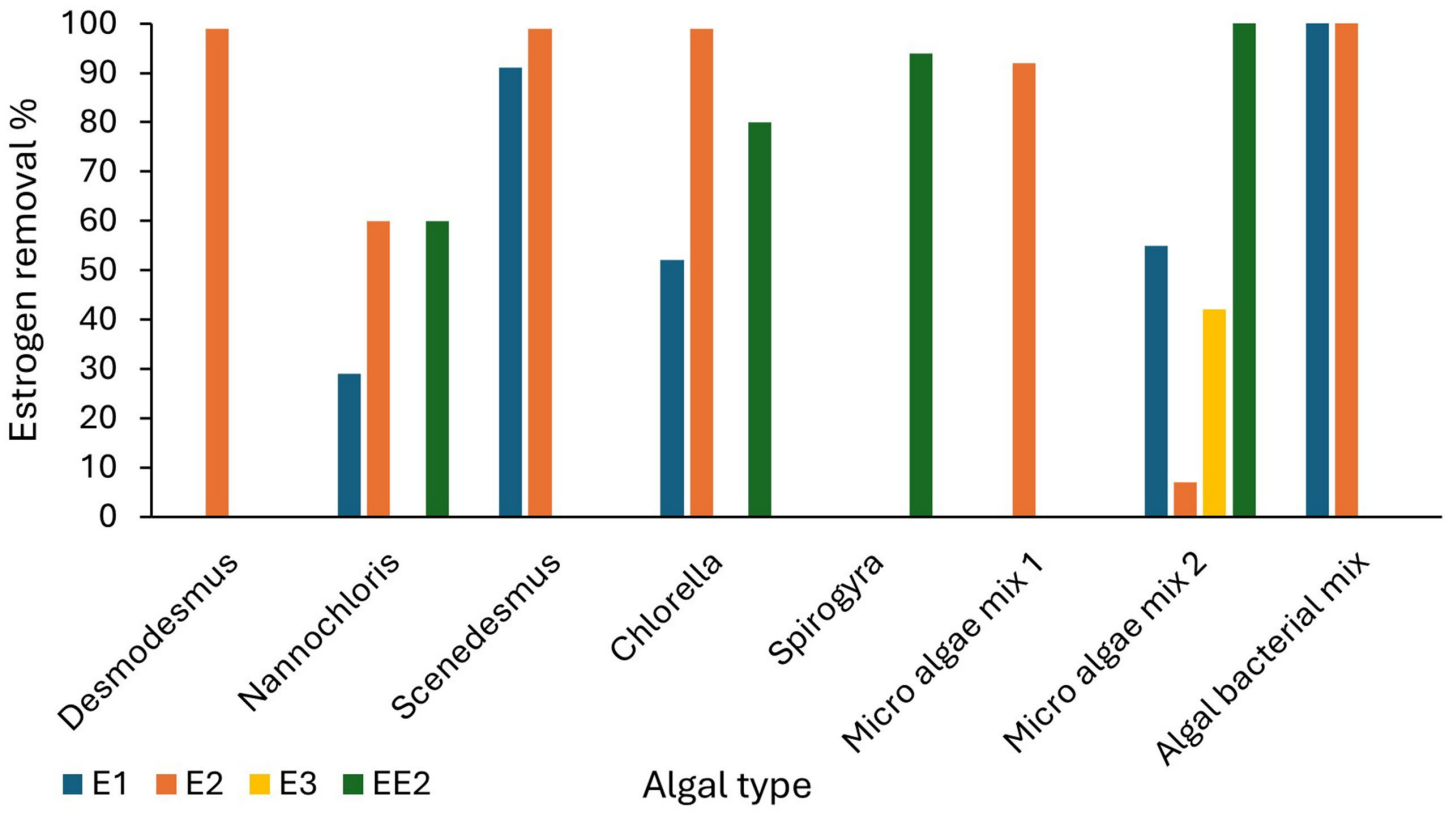
Estrogen removal efficiencies of different algal cultures (Garcia-Rodríguez et al., 2015; Bai and Acharya, 2019; Ruksrithong and Phattarapattamawong, 2019; Vassalle et al., 2020a; Wang et al., 2020; Bano et al., 2021; Prosenc et al., 2021; Pazmino-sosa et al., 2024).

Algal mixtures also have shown moderate to high removal of estrogens from wastewater. For example, Vassalle et al. (2020b) observed 55% of E1, 7% of E2, and 42% of E3 removed by micro algae from primarily treated sewage water at a range between 54 and 148 ng/L. Bano et al. (2021) observed 92% removal of E2 from spiked algal cultures at 16,000 μg/L in 12 days. An increased removal of estrogens (100% of E1 and E2) have been achieved by algal bacterial mixed cultures (Prosenc et al., 2021). Algal bacterial mix cultures have shown increased removal efficiencies (100% removal of E1 and E2) compared to *Chlorella vulgaris* (66% of E1 and 99% of E2) in a study by Prosenc et al. (2021) (Figure 5).

Filamentous algae can be used in HRAP (HRFAP-high-rate filamentous algal ponds) to remove organic pollutants from wastewater (Liu et al., 2020). Applying filamentous algae in wastewater treatment have advantages over microalgae such as high resistance to predation (Liu et al., 2016), able to withstand flow fluctuations, and the ability to increase SRT over HRT (Liu et al., 2020). They can grow in wastewater containing high organic content, nutrients and other pollutants. They easily adapt to varying environmental conditions, effectively compete with undesired species and maintain consistent biochemical composition with high areal productivity (dry weight of produced per unit area per unit time; Liu et al., 2020). On top of these factors, filamentous algae have the advantage of maintaining monocultures of the desired strain allowing selection of algal cultures that can remove specific contaminants in wastewater (Liu et al., 2020). There have only been a few reports so far on using freshwater algal monocultures for removing estrogens from wastewater. *Oedogonium*, *Cladophora*, *Spirogyra*, *Rhizoclonium*, *Microspora*, *Klebsormidium*, and *Stigeoclonium* (Liu et al., 2016) are the most used filamentous algae in wastewater treatment, but research on estrogen removal by filamentous algae is limited with only two laboratory scale studies for *Spirogyra*. Song et al. (2011) showed that free water surface treatment wetlands (FWS TWs) containing *Spirogyra* monoculture removed 42, 24, 55% of E1, E2, and EE2 from wastewater effluent, while (Garcia-Rodríguez et al., 2015) showed 94% removal of EE2 by *Spirogyra* sp. but this was in the presence of *Lemna* sp.

## 4 Estrogen removal mechanisms

Estrogen removal in both in TWs and HRAPs occurs through a combination of biotic and abiotic mechanisms (Figure 1). The main abiotic estrogen removal processes are adsorption to solid phases (substrates) and photolysis. The biotic processes include microbial processes of degradation, bioadsorption and bioaccumulation (uptake by plants and algae; Song et al., 2011; Hakk et al., 2018; Ilyas and Van Hullebusch, 2020). However, the predominant mechanism and/or the combination of the mechanisms may vary depend on the treatment system, operational conditions and environmental factors.

### 4.1 Adsorption

Estrogens are quickly and easily adsorbed onto soil and particulate organic matter (including plant litter), solid surfaces and the biofilms inhabiting them, vegetation stems and root surfaces, as well as organic colloids (Sharif et al., 2014; Ilyas and van Hullebusch, 2020). For example, the removal of E1, E2, E3 and EE2 from wastewater in TWs was found to be due to sorption to soil particles, while the removal of E1 and E2 was found to be due to adsorption to plant materials (Sharif et al., 2014; Ilyas and van Hullebusch, 2020). Estrogens are rapidly removed from the liquid phase until the sorptive capacity of the sorbent is exceeded or equilibria is attained between the liquid and organic carbon associated with the sorbent. The time to reach equilibrium depends on the properties of sorbent (high surface area and high adsorption capacity increase the rate of sorption), physicochemical properties of estrogens and operational conditions (e.g., higher ratio of sorbent volume to liquid volume increases estrogen sorption). 90% of adsorption equilibrium concentrations of estrogens in the liquid and solid phases is reached within a short period of time (de Mes et al., 2005), for example Lai et al. (2000) observed free estrogens (E1, E2, E3, EE2 and mestranol) reached an equilibrium with sediment within 0.5 h under laboratory conditions. Therefore, a significant reduction in the estrogen level in the liquid phase can be observed at the initial stages of contact with solid surfaces.

**Figure 6:**
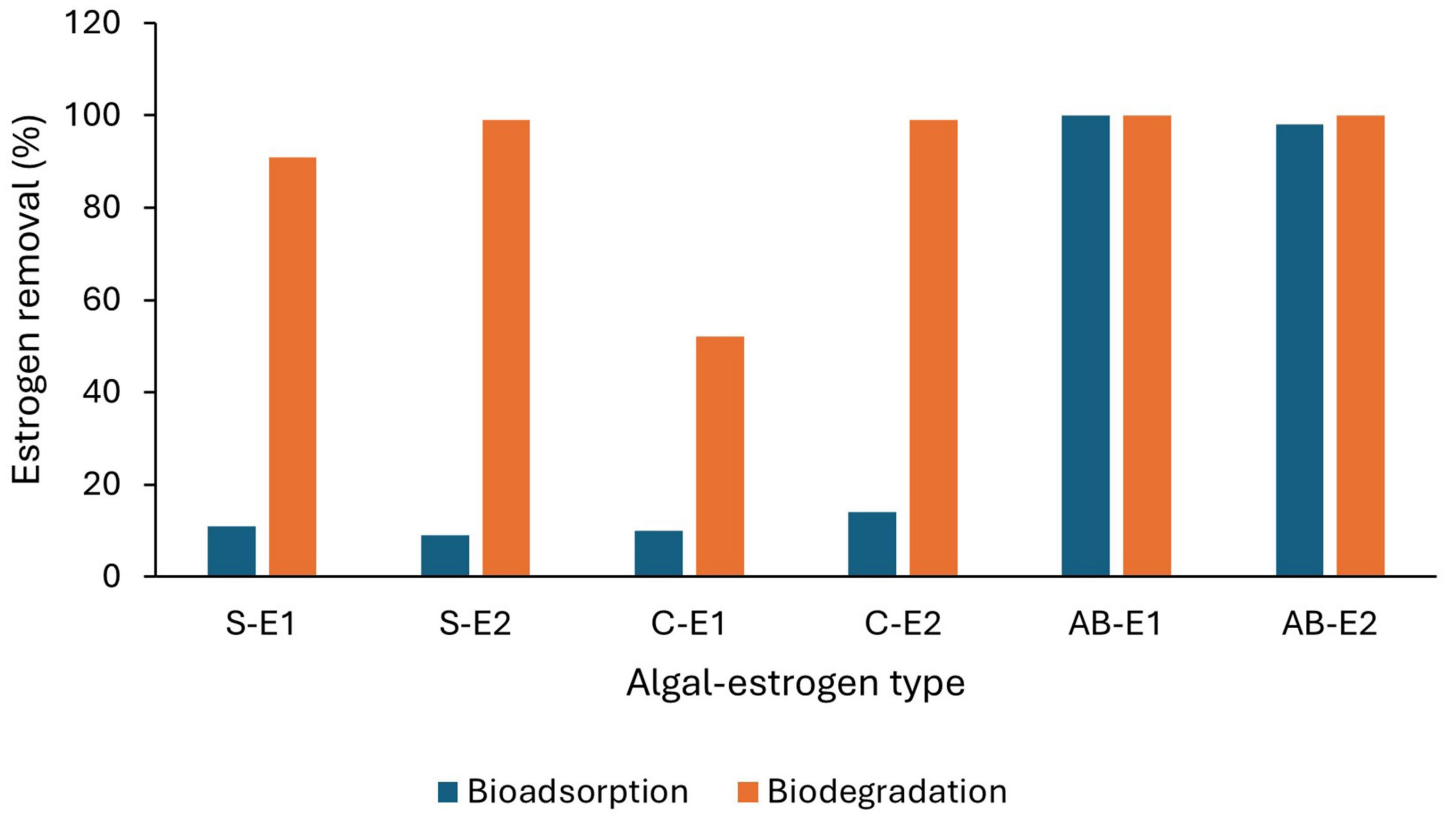
Removal efficiencies of estrogens (E1 and E2) by bioadsorption and biodegradation by algal cultures. S-*Scenedesmus*, C-*Chlorella*, AB-Algal bacterial culture (Ruksrithong and Phattarapattamawong, 2019; Prosenc et al., 2021).

Low levels of estrogen removal by bioadsorption have been reported. Solé and Matamoros (2016) observed <5% bioadsorption of E2 from secondary treated water by a mixed culture of *Chlorella* sp. and *Nitzschia acicularis* under laboratory conditions in 10 days. Ruksrithong and Phattarapattamawong (2019) observed 10% of E1 and 14% of E2 removal by *C. vulgaris* and 11 and 9% removal of E1 and E2, respectively, by *Scenedesmus obliquus* from synthetic wastewater under laboratory conditions in 6 h due to adsorption. However, (Prosenc et al., 2021) observed 100% adsorption of E1 and 97–98% of E2 with an algal-bacterial mixed culture (Table 4 and Figure 6) which may be due to bacterial biofilm on the algal surface.

### 4.2 Biodegradation

Biodegradation of estrogens is an enzyme catalysed biological process where contaminants are transformed into other products or completely mineralised (Khanal et al., 2006) by microorganisms under aerobic, anoxic, and anaerobic conditions. Bacteria, algae, and fungi are capable of biodegrading and transforming estrogens and bacteria-algae and bacteria-fungi symbiotic relationships can enhance estrogen removal in comparison to the individual microorganisms (Figures 5, 6; Matamoros et al., 2015; Pratush et al., 2020; Prosenc et al., 2021). Several bacteria and fungi with the ability to remove estrogens from sewage waste have been isolated and identified.

*Rhodococcus zopfii* and *Rhodococcusequi* bacteria found in sewage reduced E2 from 100 mg/L to 1 mg/L within 24 h (Yoshimoto et al., 2004). Other bacteria able to degrade estrogens include *E. coli, Pseudomonas fluorescens*, and *Bacillus thuringiensis* in sewage sludge (Yu and Huang, 2005) and *Cornybacterium* sp. in manure (Khanal et al., 2006). Shi et al. (2002) isolated *Fusarium proliferatum* from cowshed effluent and achieved 97% removal of EE2. Tanaka et al. (2000) showed that laccase enzyme produced by *Pycnoporus coccineus* degraded EE2 to a greater extent (75%) than E1 (40%) under laboratory conditions. Of 20 white rot fungi examined by Fujita et al. (2002), 13 strains removed either E1 or E2 or both achieving in the range of 5.5 to 99.9% removal, and *Trametes versicolor* (91% E3 and 60% E2) and *Phellinus gilvus* (>99.9% E3, 89% E2 and 46% E1) fungi have shown improved removal efficiencies compared to other fungal species.

Biodegradation of estrogens start with the deconjugation of glucuronide conjugated and sulfate conjugated estrogens. Glucuronide conjugated estrogens are easily deconjugated during sewage treatment processes due to the presence of β-glucuronidase secreted by *Escherichia coli* (Birkett and Lester, 2002), but sulfate conjugates persist longer probably due to a lack of arylsulfatase enzyme secreting bacteria (Ascenzo et al., 2003; Kumar et al., 2012) in the wastewater treatment system. Both glucuronide conjugated E2 and E1 had half-lives of 0.4 h and sulfate conjugated E1 and E2 had 13.9 h and 11.5 h half-lives, respectively, in wastewater (Kumar et al., 2012). Only 60 and 40% of glucuronide E2 and E1 and 12% of all types of sulfate conjugated estrogens were transformed into their parent forms. The remainder may presumably be either metabolised or adsorbed onto the solid phase. The mechanism of estrogen deconjugation during wastewater treatment is not well understood (Ascenzo et al., 2003).

**Figure 7:**
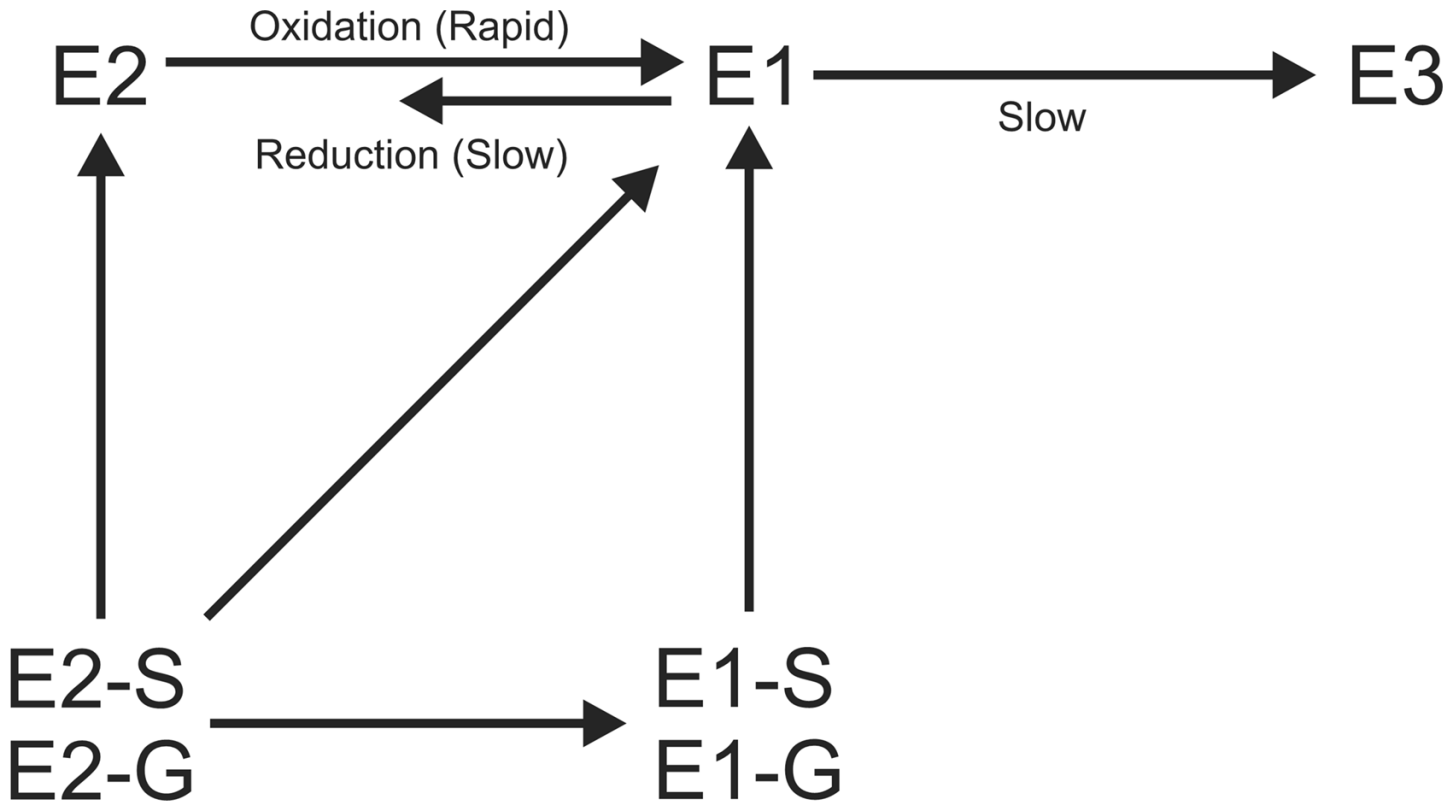
Basic biological transformation routes for estrogens. S-Sulfate conjugated, G-Glucuronide conjugated.

Free estrogens undergo further degradation after deconjugation; E2 in wastewater is rapidly biodegraded into E1(de Mes et al., 2005; Ternes et al., 1999) then slowly into E3 and other metabolites (Kozlova and Levin, 2022) and is ultimately mineralised to CO_2_ and water (de Mes et al., 2005; Figure 7). The major microbial degradation mechanisms of estrogens can either be metabolic (microorganisms use estrogens as their sole source of carbon and energy for growth) or co-metabolic (do not use estrogens as their sole source of carbon for growth but degrade them into other products; Yu et al., 2013; Pratush et al., 2020). By co-metabolism, persistent substances can be converted into potentially biodegradable products (Gröning et al., 2007; Tran et al., 2013).

Many enzymes are involved in the biodegradation or biotransformation processes of estrogens. Hydroxylase and dioxygenase are the most important enzymes that regulate steroid hormone biotransformation among major enzymes including dehydrogenase, cytochrome P450, ring-cleavage dioxygenase, hydroxylase, monooxygenase, isomerase, hydratase, and demethylase (Pratush et al., 2020). Many microorganisms with these estrogen degrading enzymes have been identified. The catabolism of E2 in *Mycobacteria, Nocardia*, and *Rhodococcus* sp. is initiated by17β-hydroxysteroid dehydrogenase and 3α-hydroxysteroid dehydrogenase enzymes (Pratush et al., 2020). 3β,17β-hydroxysteroid dehydrogenase (3β,17β-HSD, EC 1.1.1.51) catalyses the transformation of E2 to E1. E1 is transformed into 4-hydroxyestrone in the presence of estrone 4-hydroxylase enzyme (Pratush et al., 2020).

Biodegradation reduces the estrogenic potency of estrogens by transforming them into non-biologically active products (Maryjoseph and Ketheesan, 2020). Even though the degradation processes of estrogens have not been completely identified, different degradation paths have been suggested by several researchers (Ternes et al., 1999; Layton et al., 2000; Lee and Liu, 2002; de Mes et al., 2005; Czajka and Londry, 2006). The degree of biodegradation depends on various factors including the physicochemical properties of the estrogen, algal and bacterial species present, enzymatic pathway and environmental conditions (Maryjoseph and Ketheesan, 2020). A number of bacterial strains have been identified for biodegradation of E2. For example, both *Sphingomonas* sp. strain KC8 (Chen et al., 2017) and strain BHUBP7 of *Enterobacter* sp. Prakash et al. (2023) follow the dioxygenase-mediated 4,5-*seco* pathway to degrade E2 under aerobic conditions. E2 was initially transformed to E1 by dehydrogenation followed by subsequent hydroxylation and oxygenolytic degradation to form 4-hydroxyestrone (Chen et al., 2017; Prakash et al., 2023). *Sphingomonas* sp. strain KC8 transforms 4-hydroxyestrone to pyridinestrone acid but further degradation of pyridinestrone acid needs to be studied (Chen et al., 2017). *Enterobacter* sp. BHUBP7 strain transforms 4-hydroxyestrone to 3-(7a-methyl−1,5-dioxooctahydro-1H-inden-4-yl) propanoic acid (HIP) with 11 other intermediate metabolites by sequential hydrogenation, hydroxylation, and de-acetylation reactions and HIP was completely degraded following the common HIP degradation pathway (Pratush et al., 2020). *Enterobacter* sp. EE2 also degraded by BHUBP7 strain following the same pathway and with almost similar metabolites as E2 (Pratush et al., 2020).

Algae mediated biotransformation of estrogens is also possible and comparatively it is more important than other removal processes such as photolysis [<20% removal of E2 achieved by *Desmodesmus* sp. (Wang et al., 2020)] and sorption [only 15% of E1 and E2 by *Scenedesmus obliquus* and *Chlorella vulgaris* (Ruksrithong and Phattarapattamawong, 2019)].

### 4.3 Photodegradation

The removal of estrogens either by direct or indirect photodegradation is a common and important estrogen removal mechanism in HRAPs as well as in FWS TWs. Many organic compounds including estrogens absorb solar radiation below 280 nm and undergo direct photolysis (Adeel et al., 2017; Gmurek et al., 2017; Liu et al., 2021). Wastewater contains many photosensitive compounds including nitrates, nitrites, and humic substances, (Norvill et al., 2016), polysaccharides and proteins (Yang X. et al., 2018) excreted by microalgae that produce free radicals to induce photolysis of estrogens (Bai and Acharya, 2019). For example, Leech et al. (2009), observed a significant increase in E2 degradation rate in the presence of humic acid (2 mg/L), and total E2 removal was almost doubled. Therefore, half of the total E2 degraded can be attributed to the photoproduced hydroxyl radicals of humic acid.

The efficiency of estrogen removal by photodegradation is relatively low. Photolysis under laboratory conditions has also been observed, but no significant removal of E1 and E2 occurred (Zhang et al., 2014), while Wang et al. (2020) found less than 20% of E2 was removed. Similarly, Ávila et al. (2014) also observed up to 21% of EE2 removal within a FWS treatment wetland may be attributed to exposure to direct sunlight and photolysis.

### 4.4 Bioaccumulation

Bioaccumulation is an intracellular process that occurs only in viable cells and subsequent intracellular enzymatic biodegradation of estrogens may occur (Oruganti et al., 2022). However, the removal of estrogens by bioaccumulation into microalgal cells has been demonstrated to be low between 0.2 and 9.6% for E2 and EE2 in laboratory experiments (Wang et al., 2017).

## 5 Factors influencing removal of estrogens

Previous studies on estrogen removal by both TWs and HRAPs have shown that the removal efficiency of estrogen depends on many factors including, the physicochemical properties of estrogens, treatment process, organisms involved and environmental factors. The chemical structure (type of functional groups) and the physicochemical properties [hydrophobicity and hydrophilicity (Oruganti et al., 2022)] of estrogens (Table 1) influence which removal mechanisms predominate during treatment using TW and HRAPs. Operational conditions such as hydraulic loading rate (HLR), hydraulic retention time (HRT) and depth of the treatment bed/pond, ionic strength of the aqueous phase (Norvill et al., 2016), the concentration of estrogens (Priyadarshani et al., 2012; Campos et al., 2019; Sutherland and Ralph, 2019) and environmental factors such as pH, dissolved oxygen concentration, temperature, light intensity, and light dark cycle also, directly, or indirectly influence the rate of estrogen removal by TWs and HRAPs.

The low water solubilities (0.3–13.3 mg/L), partition coefficients (log K_ow_; 2.4 to 3.6) and high acid dissociation constants (pK_a_; ~10; Table 1) promote the removal of estrogens from the liquid phase into solid phases such as the filter substrates or biomass (Sharif et al., 2014; Ilyas and van Hullebusch, 2020). For example, mestranol has a lower water solubility and higher log K_ow_ (4.6) than E1 and E2 and will show a greater adsorption to sediment (4.5–5.5 μg/g) compared to E1 and E2 (3.2–4.1 μg/g; Lai et al., 2000; Table 1).

The influence of external factors on the removal of estrogens from wastewater by different mechanisms associated with TWs and HRAPs are summarized in Table 5 and discussed below.

**Table 5:** Factors influencing estrogen removal and solutions to enhance removal.

Factor		Influence on the removal mechanism			Possible solutions
		Adsorption	Biodegradation	Photodegradation	
HLR	High	Decreases adsorption due to high pollutant load and low contact time	Decreases biodegradation due to high pollutant load and low contact time	Increases photosensitizers increasing photodegradation. High organic concentration reduces the light penetration reducing estrogen removal	Reduce hydraulic loading, reduce throughput and increase residence time
	Low	Increases retention time and increases adsorption	Increases retention time and increases biodegradation		
HRT	High	Increases the contact time with the solid phases and increases adsorption	Increases the contact with microorganisms increasing removal. Allow slow growers to adapt to the system and increase biodegradation	Increases contact time increasing degradation	
	Low	Reduces adsorption due to low contact time	Reduces biodegradation due to low contact time	Decreases Photodegradation due to low contact time	
Water level/bed depth	High	Reduces temperature and increases adsorption	Decreases DO concentration, light intensity and temperature, decreasing aerobic degradation of estrogens	Reduces light penetration and reduces photodegradation	Maintain the pond depth to a level that provide the optimum level of oxygen, temperature and light intensity require for maximum estrogen biodegradation depending on the season Increase in depth of treatment bed and the water level and depth of algal ponds
	Low	No significant effect	Increases biodegradation	Favorable for light penetration and increases photodegradation	
	Low	Decrease (bio)adsorption	Decreases biodegradation	No significant effect	
Dissolved oxygen concentration	High	No significant effect	Provide aerobic conditions favorable for biodegradation	Formation of oxygen free radicals increases photodegradation	Maintain aerobic conditions >4 mg/L O2, reduce biological oxygen demand
			Supersaturation of oxygen in algal ponds reduces CO_2_ concentration and increase pH reducing estrogen removal		Add carbonates, Increase HLR and reduce HRT, Vertical mixing, Maintain biomass
	Low		Reduces aerobic biodegradation	Reduces photodegradation	Pulse loading in TWs, Reduce water level/depth, Aeration
pH	Low	Increases ionization of estrogens, increasing solubility and reduce adsorption	pH unfavourable for microorganisms reduce degradation	Increase in photosensitizers increase photodegradation	Dose with acid or base or increase buffering capacity to maintain neutral pH
	Low	Favors adsorption			
Ionic strength	High	Increases binding free estrogens to substrates	No significant effect	Increase in photosensitizers increase photodegradation	Maintain the ionic strength favorable for adsorption. E.g. maintain favorable nitrate concentration
	Low	Increase binding conjugated estrogens to substrates			Maintain HLR and keep estrogen level proportionate to the solid phases that adsorb estrogens
Biomass	High	Increases (bio)adsorption	Increases biodegradation	Decreases light penetration and reduces photodegradation	Maintain HLR, HRT and organic loading to maintain a biomass favorable for estrogen removal
	Low	Decrease (bio)adsorption	Decreases biodegradation	No significant effect	
Physicochemical properties of estrogen; Solubility Hydrophobicity Partition coefficient pK_a_	Low	Favors adsorption of conjugated estrogens	Effects the adsorption, affecting biodegradation	No significant effect	Adjust pH to Favor adsorption Maintain conditions favourable for hydrophobic interactions
	High	Favors adsorption of free estrogens			
Physicochemical properties of substrate materials	Organic content	Increases adsorption	Effects the adsorption, affecting biodegradation	Not applicable favour	Increase organic content in the filter substrates
	porosity				Use porous materials or mix porous material with other filter substrates
	Hydrophobicity				Reduce substrate particle size or use substrates materials with low particle size
	Low particle size				Adjust pH to favor adsorption, increase ionic strength of solution to drive hydrophobic interactions
Concentration of organic and suspended particulate matter	High	Increases competition for binding sites and reduces adsorption	Increases microbial biomass, organic matter consumption and degradation of estrogen	Increases photosensitizers increasing photodegradation. High organic concentration reduces the light penetration reducing estrogen removal	Maintain proper organic loading (HLR and HRT) favorable for estrogen removal
	Low	Reduces competition with other suspended particles and Increases adsorption of estrogens to binding sites	Reduces estrogen removal	Favorable for light penetration and increases photodegradation	
Seasonal changes	Summer	Reduces adsorption	Temperature increases in summer increases biodegradation	Increases formation of photosensitizers increases photodegradation	Adjust depth or throughput to reduce temperature changes Increase HLR and HRT
	Winter	Favorable for adsorption	Decreases biodegradation	Reduces photodegradation	Reduce throughput, Reduce water level, pond/bed depth Reduce HLR and increase HRT
Light intensity	High	Increases temperature decreasing adsorption	Increases photosynthesis and DO concentration, temperature and enhances aerobic biodegradation	Increases as chemical reactions are increased	Increase light intensity by maintaining pond depth, algal biomass and suspended materials
	Low	Increases adsorption	Decreases biomass and biodegradation	Reduces photodegradation	
Temperature	High	Increases solubility decreasing adsorption	Enhances enzymatic reactions increasing degradation	Increases as chemical reactions are increased	Maintain temperature that favors adsorption by controlling depth
	Low	Increase adsorption	Decreases biodegradation	Reduces photodegradation	
Estrogen concentration	High	Increases adsorption	Enhances enzymatic reactions and increases biodegradation	Increases estrogen removal	Maintain favorable HLR and HRT
	Low	Decreases adsorption	Decreases biodegradation	Decreases estrogen removal	

**Figure 8:**
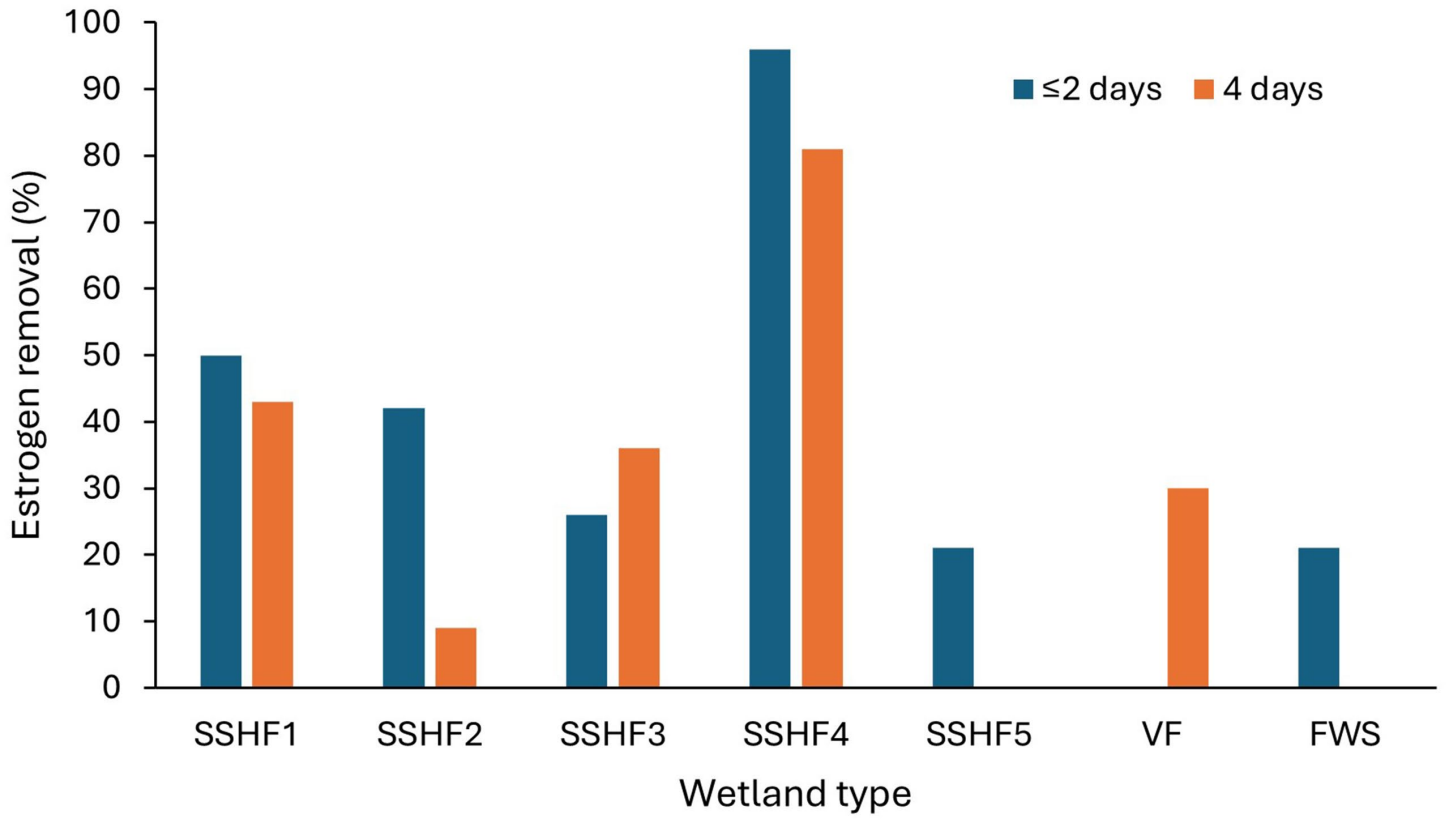
The removal efficiency of EE2 treatment wetlands at different HRTs. SSHF-Subsurface horizontal flow, VF-Vertical flow, FWS-Free water surface (Campos et al., 2019; Ávila et al., 2014).

**Figure 9:**
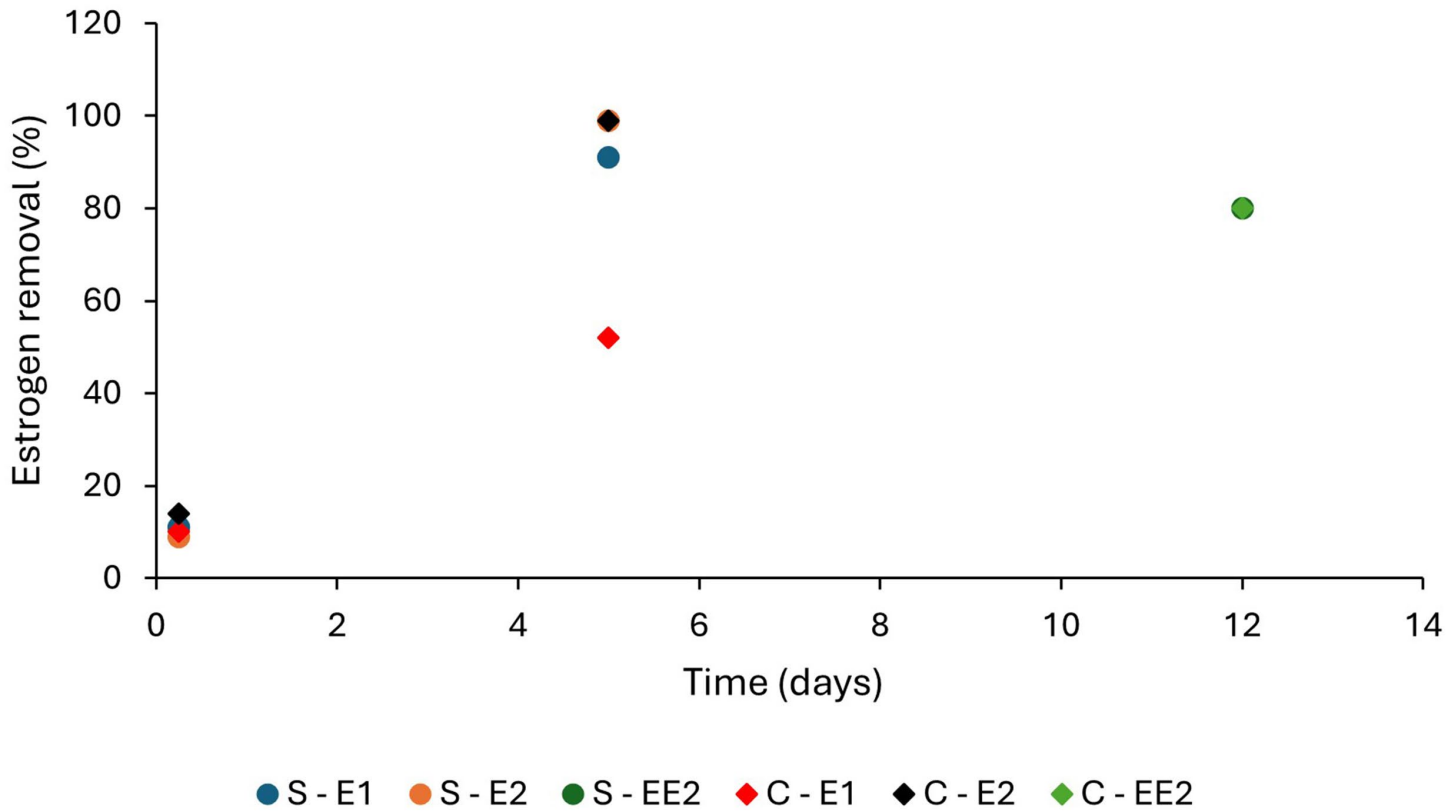
The removal efficiencies of estrogens by algae at different HRTs (Ruksrithong and Phattarapattamawong, 2019; Pazmino-sosa et al., 2024; S, *Scenedesmus*; C, *Chlorella*).

Both HLR (flow rate per unit area) and HRT have considerable impact on adsorption of estrogens from the liquid phase, as these parameters decide the organic loading and contact time of estrogens with solid surfaces (Dai et al., 2016). Increased HLR reduces HRT and increases organic loading. Therefore, removal of estrogens by adsorption can be expected to decrease due to a shorter contact time, increased competition for adsorption sites by the organic matter and reduced oxygen availability. Ávila et al. (2014) observed a decrease in removal of EE2 in treated wastewater from 25 to 15% when HLR was increased from 133 to 367 L/m^2^/d. Increased HRT also can inversely affect the removal. Campos et al. (2019) observed a reduction in EE2 removal with the increase in HRT from 2 to 4 days (Figure 8) for four SSHF TWs consisting of different substrates and macrophytes. A reason was not given but it could have been due to either a reduced dissolved oxygen content or a lower nutrient supply for the HRT of 4 days compared to the HRT of 2 days. Ruksrithong and Phattarapattamawong (2019) observed an increase in the removal of E1 (from 11 to 91%) and E2 (from 9 to 99%) from synthetic wastewater under laboratory conditions when the retention time increased from 6 h to 5 days (Table 4 and Figure 9).

The pH value of the wastewater in the treatment system influences the ionic state of both estrogens and the solid substrates which significantly influences the partitioning of estrogens to solid phases (Kumar and Mohan, 2011). As the pK_a_ values of estrogens are high, the dissociation of hydrogen ions from estrogens occurs at high pH values, the phenolic functional groups become negatively charged (Fredj et al., 2015) resulting in desorption from the solid phase and increased solubility in the liquid phase (de Mes et al., 2005). At low pH values, estrogens have a neutral charge, exhibit low water solubilities and are readily adsorbed to solid phases. Kumar and Mohan (2011) observed 95.4% of EE2 adsorbed onto activated carbon at pH 7 under laboratory conditions and no adsorption occurred at pH 10. Similarly, Zheng et al. (2016) observed an increased adsorption of E2 to sludge at low pH, and adsorption decreased with the increase in pH. However, desorption is unlikely under normal conditions as the pH of wastewater is generally neutral. The high daytime pH (up to 11) that can occur in HRAP due to uptake of CO_2_ in the form of carbonic acid from the water for algal photosynthesis (Craggs et al., 2014) will greatly reduce estrogen removal by adsorption as the solubility of estrogens increases at higher pH.

The adsorption of estrogen to organic matter or substrate is affected by the ionic strength of the aqueous solution (Aksu and Akın, 2010; Horsing et al., 2011; Zheng et al., 2016). The increase in cation concentration in the medium neutralizes the negative charge of phenolic groups on the estrogenic steroids, and similarly, the polar acidic and phenolic functional groups on the organic matter of solid phases are also neutralized, thereby reducing the polarity and increasing the hydrophobic nature of organic matter, which will increase the sorption of estrogenic steroids. The presence of nitrate ions in biologically treated wastewater also influences E2 removal. Zheng et al. (2016) observed increased sorption of E2 to activated sludge until the ionic strength of the medium was increased up to 0.4 mol/L followed by a sharp decrease thereafter. The increase of flocculation and aggregation of suspended particles by increasing ionic strength followed by sedimentation also increases the removal of estrogen from the liquid phase (Lai et al., 2000).

The impact of temperature on adsorption of estrogen in TW is rarely reported (Song et al., 2009) and the effects are not consistent. Kumar and Mohan (2011) observed the adsorption of EE2 on to activated carbon increased up to 30°C under laboratory conditions and then decreased gradually. Dai et al. (2016) found adsorption of E2 and E1 to gravel increased from 48 to 60% and from 30 to 60%, respectively, when the temperature was decreased from 28–30°C to 12–15°C under laboratory conditions. The initial concentration of estrogen also influences the removal efficiency. Campos et al. (2019) observed an increased removal of EE2 when the initial concentration was increased from 15.6–17.6 μg/L to 94.0–109.6 μg/L.

Biodegradation of estrogens is faster under aerobic conditions in both aqueous and solid phases (Carballa et al., 2007; Song et al., 2009; Paterakis et al., 2012; Vymazal et al., 2015; Ting and Praveena, 2017). Joss et al. (2004) observed 3–5 times higher degradation of E1 when redox conditions were changed from anaerobic to anoxic and from anoxic to aerobic conditions in sludge than remained in anaerobic conditions. E2 also degrades under all redox conditions in sludge but degrades slowest under anaerobic conditions (Joss et al., 2004; Lee and Liu, 2002). The conversion of E1 back to E2 is also possible (Dai et al., 2016). Czajka and Londry (2006) observed two thirds of E2 was oxidized to E1 with no or few other metabolites, while no transformation of E2 to E1 occurred in sterile sediments. EE2 does not biodegrade as readily as E2 (Ternes et al., 1999; Ting and Praveena, 2017) and sometimes does not biodegrade at all (de Mes et al., 2005). The differences in degradation rates can be related to the structural differences of estrogens. EE2 has both a hydroxyl group and an ethynyl group at the same carbon which hinders microbial attack (Manickum and John, 2014; Silva et al., 2012). Joss et al. (2004) and Czajka and Londry (2006) found EE2 only in sludge under aerobic conditions.

If organic matter is limited for estrogen metabolism, increased HLR supplies more substrate for microorganisms, therefore the removal of estrogens can increase. Sharif et al. (2014) increased removal of E2 from 1.8 to 2.5 g/ha-d by increasing carbon loading rate (CLR) from 6 × 10^3^ kg-DW/ha-yr to 1.2 × 10^4^ kg-DW/ha-yr by keeping the HLR at its highest (20 cm/day). Increased HRT allows greater interaction between estrogens and microorganisms increasing the biodegradation rate and increased estrogen removal (Qiang et al., 2013). Longer HRTs also allow the growth of more specific and diverse microorganisms enabling them to adapt to the conditions in treatment process (Koh et al., 2008). Sharif et al. (2013) observed an increase in the removal of E2 from 56, 66, and 70% with the increase of HRT from 2.1 to 2.6 days and to 4.8 days, respectively. However, increase in HLR and HRT do not always increase the estrogen removal efficiency. Campos et al. (2019) found removal of EE2 decreased from 95.6 to 80.7% with an increase in HRT from 2 to 4 days.

Theoretically, biodegradation of estrogens could increase with increasing temperature as both biological and chemical degradative reactions are enhanced by increasing temperature (Zeng et al., 2009). Therefore, relatively high removal efficiencies of estrogens can be expected in the summer as the efficacy of enzymatic reactions increase with temperature (Maryjoseph and Ketheesan, 2020; Matamoros et al., 2015; Oruganti et al., 2022). However, the results reported so far on the effect of temperature on estrogen removal are contradictory. Zeng et al. (2009) reported a rapid aerobic biodegradation of E2 at temperatures between 20 and 30°C. Layton et al. (2000) did not observe a significant difference in the removal of E2 at low temperatures (5–10°C). Koh et al. (2008) reported higher removal of E2 during summer (87%) than in winter (70%), but the difference was not significant. Dai et al. (2016) observed higher removal of both E1 (55–90%) and E2 (46–81%) in summer than in winter [E1 (24–61%) and E2 (2–43%)]. The deconjugation of estrogens was increased with higher temperature and, Dai et al. (2016) and Zheng et al. (2016) observed a higher deconjugation rate of sulphate conjugated E1 in summer than in winter. However, Koh et al. (2008) reported a lower reduction in sulphate conjugated E1 and E2 during summer than in the winter although the reasons were not explained.

The high rates of algal photosynthesis during the daytime in summer produce high concentrations of dissolved oxygen (Craggs et al., 2004) and during peak solar radiation, dissolved oxygen concentrations can reach supersaturation typically to 200–300% (Craggs et al., 2014; Norvill et al., 2016). Due to high consumption of CO_2_, pH levels can reach as high as 11 (Craggs et al., 2014; Norvill et al., 2016). Many microalgae can grow in a broad pH range (7–9), with maximum growth at pH between 8.2 and 8.7 (Oruganti et al., 2022). Supersaturation of oxygen and high temperature and pH can cause photoinhibition which limits algal productivity (Weissman et al., 1988; Kong et al., 2010). However, this can be overcome by using available bicarbonates to reduce pH changes (Craggs et al., 2014).

Increasing depth of the treatment beds/ponds reduces DO concentration, redox potential, light intensity, and temperature (García et al., 2005; Dwire et al., 2006; Song et al., 2009), affecting microbial consortia (Meng et al., 2019) and their density (Jebali et al., 2018). At greater depths, only anoxic and/or anaerobic biodegradation takes place reducing estrogen removal. Tietz et al. (2007) showed there was a higher microbial density within the top 10 cm of the media in a SSVF TW due to elevated DO concentration. Song et al. (2009) observed increased removal of E1, E2 and EE2 (64.9, 54.9 and 39.2% respectively) in a 30 cm deep SSVF compared to a 60 cm deep SSVF (61.5, 47.1 and 38.9% respectively) in a field study. Ávila et al. (2014) achieved 21% removal of E1 in SSHF (30 cm deep), similar to a 10 cm deep FWS, may be due to the increased surface area for adsorption with increased bed depth or transformation of E1 to other products.

A greater pond depth reduces the photolysis of estrogens as photolysis mainly depends on light intensity (Kushwaha et al., 2024). Photolysis also depends on the initial concentration of estrogens, pH, composition and temperature of the water (Gmurek et al., 2017). High HLR increases the organic matter content of the water, reducing light penetration, thereby decreasing estrogen removal. Sharif et al. (2014) observed a significant reduction in photolysis of E2 with increasing organic matter content in water under laboratory conditions. Ávila et al. (2014) estimated up to 21% of EE2 removal within a FWS treatment wetland may be attributed to exposure to direct sunlight and photolysis.

Algal growth is affected by biological factors such as competition between species, grazing by invertebrates and viral infections (Grobbelaar, 2000; Larsdotter, 2006; Park et al., 2013) which are influenced by operational parameters such as organic loading rate, depth, HRT, and horizontal mixing velocity (de Godos et al., 2012; Craggs et al., 2014; Quijano et al., 2017) which will also affect the estrogen removal.

## 6 Improving estrogen removal

Research done so far on estrogen removal by TWs and HRAPs have shown that both systems perform equally or better in estrogen removal than traditional wastewater treatment techniques. Estrogen removals in TWs have been reported as high as 85% for E1 (Vymazal et al., 2015; Herrera-Melián et al., 2018), >80% for E2 (Vymazal et al., 2015), 100% for E3 (Herrera-Melián et al., 2018) and > 98% for EE2 (Chen et al., 2020; Table 3). HRAP has shown up to 100% removal of both E1 and E2 from wastewater (Prosenc et al., 2021) and 97% removal of EE2 from treated wastewater under laboratory conditions (Solé and Matamoros, 2016; Table 4). The factors limiting the removal efficiencies have been identified and possible alternatives have also been introduced. For example, hybrid systems, artificial aeration, recirculation, or reciprocation (Dotro et al., 2017) and, novel substrate materials with increased adsorption have been applied to improve estrogen removal efficiencies in TWs. Microorganisms (bacteria, algae and fungi) with efficient estrogen degradation have been isolated and identified. However, optimum operational conditions or the best TW or HRAP system for estrogen removal have not been identified so far and more research on increasing the removal of estrogen from wastewater is highly required.

Enhancing and maintaining the positive influences and overcoming the negative influences on removal of estrogens from wastewater in the field is challenging as estrogen removal depends on multiple variables. The physicochemical parameters of estrogens that influence on removal are inherent and cannot be changed and environmental parameters such as temperature, dissolved oxygen, and light dark cycles are hard to control, adjusting operational parameters of the treatment process is the most feasible approach to improve removal of estrogens from wastewater.

Maintaining DO concentration is important for aerobic biodegradation to take place. Several techniques have been introduced to maintain aerobic condition in TWs. The use of artificial aeration increases the oxygen transfer rate (Pascual et al., 2024). In addition, various reoxygenation approaches have been developed such as effluent recirculation (Vymazal et al., 2021). Therefore, the aerated TW system is a promising technology for removing estrogens from wastewater (Pascual et al., 2024) but, artificial aeration adds additional operational cost. Designing and identifying the factors to optimizing the aeration to ensure maximum O_2_ transfer from the gaseous to the liquid phase with a low cost is in vital importance (Pascual et al., 2024). Freeman et al. (2018) showed that, aeration efficiency significantly depends on the aeration rate and increase in aeration rate decreases the aeration efficiency. Therefore, the optimization of the aeration rate is of vital importance as it has a direct impact on energy consumption which affect the sustainability TWs. Aeration in TWs prevents solids accumulation even at higher loading rates, indicating low risk of clogging and, also reduces the area requirement for HSSF TWs (Pascual et al., 2024).

As estrogens are easily adsorbed to solid phases, promoting adsorption at the initial step of treatment process followed by biodegradation can be applied to optimize estrogen removal using TWs. In SSUVF, wastewater will initially flow through a region with reduced or no DO, low temperature and no light as the flow direction is upwards. Therefore, adsorption of estrogen reduces the estrogen concentration in the liquid phase. As wastewater reaches the top layer of the TW receives sunlight and has a high DO concentration, estrogens may undergo aerobic biodegradation and photodegradation, increasing removal, therefore, SSUVF TW may perform better than SSDVF. Enhanced estrogen removal is possible in passing wastewater first through SSUVF TW followed by FWS. Similar performance may be achieved by combining both units (SSUVF TW in bottom and FWS on top) together to form IMWF, which reduces the area footprint while achieving higher estrogen removal. IMWF is a recent development in TWs as several units of VF TWs can be stacked up to form a single IMWF with a lower footprint which achieves pollutant removal efficiencies like multiple VF TW units in series. However, IMWF have not been assessed for their efficacy to remove estrogenic steroids from wastewater. As IMWF have shown increased removal efficiencies of nutrients and organic pollutants such as pharmaceuticals, IMWF should also exhibit improved estrogen removals from wastewater compared to other TW systems.

Removal of estrogens by adsorbing into substrates used in TW is an efficient, effective and economic technique (Bilal et al., 2022). TWs have the advantage of using substrate from a wide range of sources including various rocks (sand, gravel) and minerals (zeolite), food wastes, plant materials and construction wastes. Substrates rich in organic matter have shown increased removal of estrogens from wastewater. Herrera-Melián et al. (2018) achieved higher removal of E2 and E3 (100 and 31% respectively) with mulch than with gravel (71% and −53% respectively). Mixing traditional filter materials (sand and gravel) with benign and economically feasible organic materials such as wood chips, wood bark, saw dust, fruit and vegetable wastes, trimmings from kiwifruit and grape vines and grapes used for wine productions would increase estrogen removal, but require extensive research. Biochar produced from wood is also a good alternative with increased adsorption capacity. Campos et al. (2019) achieved 46% greater removal of EE2 from synthetic wastewater, with gravel and bamboo charcoal mixture than with gravel only under laboratory conditions. As estrogens are moderately hydrophobic, substrates with hydrophobic surfaces such as plastics will enhance the adsorption but poses the risk of releasing toxic chemicals to the environment as well of breaking down into microplastics, which is also considered an emerging contaminant (Chen et al., 2020). IMWF has the advantage of using multiple substrates in one unit that provides the potential to improve estrogen removal from wastewater. Estrogen adsorption can be enhanced by maintaining neutral pH of the aqueous phase by dosing with acid or base, or by increasing the buffering capacity. The ionic strength of the aqueous phase can be manipulated, e.g., nitrate concentration, so it is favorable for adsorption.

Maintaining a low HLR and increasing HRT should improve estrogen removal due to increased contact time for adsorption and biodegradation, but this will depend on the rate of adsorption which will depend on the surface area of the substrate and the adsorption favorability. For example, good removals using some substrates can be achieved at high estrogen concentrations, but the same substrate gives poor or negligible removal at low estrogen concentrations due to the adsorption not being favorable, so the HRT could be as high as possible or there could be as much substrate as possible, but the system as a whole could never completely remove all the estrogens. Likewise, if the adsorption rate is high, increasing the bed depth or HRT is not likely to give additional benefit. For example, Song et al. (2009) achieved similar removal efficiency for EE2 in VF TW when the bed depth was increased from 30 cm to 60 cm and the retention time was increased from 12 to 25 h (Table 3). Song et al. (2009) also had removals of 43 to 82% in VF TW with low bed depths of 8 cm and at a low HRT of 3 h.

Clogging of the substrates in TWs will reduce their performance (Bai et al., 2016). The clogging can be overcome by replacing the substrates, but frequent replacement is not economically feasible. However, the replacement cost can be reduced by using substrate materials that can be reused (de Matos et al., 2018) or by using substrates with higher adsorption capacities. In addition, the clogging can be kept to a minimum by including a pre-treatment unit such as a septic tank, primary sedimentation tank or up-flow anaerobic sludge blanket (UASB) reactor prior to using a TW (Moreira and Dias, 2020) or by recirculating or backflushing with treated effluent (Ávila et al., 2017).

The performance of HRAP is affected by environmental factors such as seasonal variations, weather changes, diurnal light, and temperature fluctuations as well the algal consortium (Jebali et al., 2018), but increased estrogen removal and HRAP performance can be achieved by adjusting operation conditions such as pond depth, HLR and HRT rates (Green et al., 1996; Buchanan, 2014). The light penetration can be manipulated by modifying pond depth and algal concentration according to season (Park et al., 2011). For example, during the summer when light intensity is high and temperatures are warm, oxygen supersaturation and increased pH can occur due to photosynthesis by algae which reduces estrogen adsorption and biodegradation. This can be prevented by increasing HLR and reducing HRT to reduce DO and maintain the pH close to neutral, or by increasing pond depth to reduce overall light penetration to limit photosynthesis. If stratification is occurring where the top layer is supersaturated and warm while the bottom layer is anoxic and cold, including a paddlewheel to promote vertical mixing between layers will help even out the temperature, DO and pH (Acién et al., 2016; García et al., 2006). This increased mixing will also help increase CO_2_ exchange between the atmosphere and the water which will also help increase the carbonic acid concentration in the water, reducing pH. Alternatively, passing the supersaturated water with increased pH through a wetland system with a substrate rich in carbonates (e.g., crushed seashells) may be a possible solution to reduce both pH and oxygen concentration. During the winter, pond depth and algal concentration can be reduced to improve light intensity and HRT can be increased to increase the amount of time available for biodegradation, adsorption and photolysis. Alternatively, maintaining high concentrations of photosensitizers such as nitrates will increase the degradation of estrogens.

Bioaugmentation is another potential technique to improve estrogen removal in TW (Parladé et al., 2018). Addition of specific microorganisms can alter the composition, and the activity of natural biofilms present and enhance the degradation of estrogens (Iasur-kruh et al., 2011). Hom-Diaz et al. (2015) achieved 88–100% removal of E2 using *Scenedesmus* sp. and Parladé et al. (2018) obtained faster removal of E2 from urban wastewater when concentrations of naturally occurring microorganisms were increased. Iasur-kruh et al. (2011) increased the removal of E2 and E1 under laboratory conditions by bioaugmenting biofilms with EDB-LI1 bacteria isolated from TWs.

Changing algal species in HRAPs could also improve estrogen removal. For example, the use of filamentous algae in HRAP is a novel trend in wastewater treatment, however, studies on estrogen removal using filamentous algae in wastewater has been limited only to *Spirogyra* under laboratory conditions in a few studies which have shown estrogen removals of up to 94% (Garcia-Rodríguez et al., 2015). Therefore, further research on *Spirogyra* and other filamentous algae on their ability to remove estrogens from wastewater and how to optimise their performance in HRAPs will be important in further enhancing the removal of estrogens from wastewater in HRAP systems.

The efficiency of estrogen removal is assessed by measuring the disappearance of the parent molecule of estrogen. As estrogens can be transformed into intermediate products that are more harmful than the estrogens during degradation, a comprehensive understanding on removal mechanisms are essential to improve the removal efficiency (Bilal et al., 2022) because the intermediate products, end products and byproducts of estrogen degradation depends on the method of degradation. Studies on applying biosensors to track estrogens in the sub compartments (substrates, biofilms, macrophytes) will be useful to determine the removal pathways and mechanisms of estrogen in TWs and HRAPs.

The improvements that have been introduced to TWs and HRAPs so far were mainly based on the extensive experimental work at laboratory, pilot or full scale (Ilyas and Rousseau, 2024). Incorporation of the knowledge gained from practical work into mathematical and computer models such as STELLA (Ilyas and Rousseau, 2024; Kushwaha et al., 2024) will reduce the time and cost required for subsequent practical work.

## 7 Conclusion

Estrogens are problematic in wastewater discharges because they are biologically active at extremely low concentrations and most conventional treatment processes are not designed to efficiently remove them. TWs and HRAPs are economically feasible alternatives for decentralized wastewater treatment for estrogen removal.

Estrogen removal mechanisms in these systems include photolysis, adsorption, bioaccumulation, and biodegradation. Adsorption is one of the main mechanisms for estrogen removal in TWs and can be enhanced using alternative media with a high organic carbon content such as palm mulch, biochar, instead of traditional substrates such as sand and gravel. Biodegradation is another key but slow estrogen removal mechanism in TW and occurs primarily under aerobic conditions, and therefore in the unsaturated zones in wetlands. Using substrate that increases rate of estrogen adsorption in the unsaturated aerobic zone enables good removal of estrogens at high hydraulic loadings and low retention times and allows attached biomass in the unsaturated zone time to biodegrade the adsorbed estrogens. Intensified TWs with artificial aeration, recirculation and reciprocation enhance the removal of estrogens. Using IMWF allows the use of unsaturated and saturated zones for nutrient removal. Incorporating filamentous algae into HRAP systems is a promising area of future research for enhancing HRAP performance in estrogen removal and the effect of operational conditions during summer and winter on HRAP performance using filamentous algae needs to be further explored.
